# Ferroptosis Induction in Multiple Myeloma Cells Triggers DNA Methylation and Histone Modification Changes Associated with Cellular Senescence

**DOI:** 10.3390/ijms222212234

**Published:** 2021-11-12

**Authors:** Emilie Logie, Bart Van Puyvelde, Bart Cuypers, Anne Schepers, Herald Berghmans, Jelle Verdonck, Kris Laukens, Lode Godderis, Maarten Dhaenens, Dieter Deforce, Wim Vanden Berghe

**Affiliations:** 1 Laboratory of Protein Science, Proteomics and Epigenetic Signaling (PPES) and Integrated Personalized and Precision Oncology Network (IPPON), Department of Biomedical Sciences, University of Antwerp, 2610 Wilrijk, Belgium; emilie.logie@uantwerpen.be (E.L.); herald.berghmans@uantwerpen.be (H.B.); 2 Laboratory of Pharmaceutical Biotechnology, Proteomics and Mass Spectrometry Department, Ghent University, 9000 Ghent, Belgium; Bart.VanPuyvelde@UGent.be (B.V.P.); maarten.dhaenens@ugent.be (M.D.); dieter.deforce@ugent.be (D.D.); 3 Biomedical Informatics Network Antwerp (Biomina), Department of Computer Science, University of Antwerp, 2610 Antwerp, Belgium; bart.cuypers@uantwerpen.be (B.C.); kris.laukens@uantwerpen.be (K.L.); 4 Center of Medical Genetics, University of Antwerp & Antwerp University Hospital, 2650 Edegem, Belgium; anne.schepers@uantwerpen.be; 5 Center for Environment and Health, Department of Public Health and Primary Care, KU Leuven, 3000 Leuven, Belgium; jelle.verdonck@kuleuven.be (J.V.); lode.godderis@kuleuven.be (L.G.); 6 IDEWE, External Service for Prevention and Protection at Work, 3001 Heverlee, Belgium

**Keywords:** ferroptosis, multiple myeloma, DNA methylation, iron, histone post-translational modifications, epigenome

## Abstract

Disease relapse and therapy resistance remain key challenges in treating multiple myeloma. Underlying (epi-)mutational events can promote myelomagenesis and contribute to multi-drug and apoptosis resistance. Therefore, compounds inducing ferroptosis, a form of iron and lipid peroxidation-regulated cell death, are appealing alternative treatment strategies for multiple myeloma and other malignancies. Both ferroptosis and the epigenetic machinery are heavily influenced by oxidative stress and iron metabolism changes. Yet, only a limited number of epigenetic enzymes and modifications have been identified as ferroptosis regulators. In this study, we found that MM1 multiple myeloma cells are sensitive to ferroptosis induction and epigenetic reprogramming by RSL3, irrespective of their glucocorticoid-sensitivity status. LC-MS/MS analysis revealed the formation of non-heme iron-histone complexes and altered expression of histone modifications associated with DNA repair and cellular senescence. In line with this observation, EPIC BeadChip measurements of significant DNA methylation changes in ferroptotic myeloma cells demonstrated an enrichment of CpG probes located in genes associated with cell cycle progression and senescence, such as Nuclear Receptor Subfamily 4 Group A member 2 (NR4A2). Overall, our data show that ferroptotic cell death is associated with an epigenomic stress response that might advance the therapeutic applicability of ferroptotic compounds.

## 1. Introduction

Multiple myeloma (MM) is an incurable adult blood cancer characterized by uncontrolled growth of plasma cells within the bone marrow. More than 30,000 adults are diagnosed with MM every year, making it the second most common hematological malignancy after non-Hodgkin lymphoma [1]. Although therapeutic advancements over the last few decades have significantly improved the prognosis and progression-free survival of MM patients [2], the high relapse rate remains a major challenge to improve treatment outcome. In most cases, the disease is characterized by a repeating pattern of remission and relapse periods during which patients receive multiple different treatment regimens, ultimately resulting in complete treatment failure. The need to find alternative therapeutic strategies to overcome (acquired and primary) therapy resistance therefore remains a pressing matter. Myelomagenesis is frequently promoted by primary and secondary (epi)mutational events. Besides the well-catalogued genetic alterations, including chromosomal translocations and hyperdiploidy, epigenetic aberrations such as changes in DNA methylation [3,4], histone modifications [5,6], and abnormal microRNA (miRNA) expression [7,8] have been linked to MM pathogenesis. Interestingly, several studies have shown that epigenetic alterations can mediate drug resistance [9,10,11] and that rewriting the abnormal epigenetic code in MM, i.e., by epigenetic drugs, might offer novel therapeutic options [12,13]. Epigenetic compounds, including decitabine, 5-aza-2′-cytidine, and vorinostat, have indeed demonstrated potent anti-myeloma activity [14,15,16].

Ferroptosis is a form of regulated cell death (RCD) that is currently being explored as a therapeutic strategy for treatment of malignant tumors, including B-cell malignancies [17,18,19]. In contrast to other modes of RCD, such as apoptosis, ferroptosis relies on intracellular increases in free iron levels and lipid peroxidation to promote cell death [20]. Given that most tumors already exhibit high basal oxidative stress levels and an altered iron metabolism due to their increased proliferation capacity, ferroptosis induction might further be exploited to exhaust the tumoral antioxidant defense mechanisms and overcome multi-drug resistance [21]. Intriguingly, although several genes (e.g., SLC7A11, GPX4, HMOX1, ACSL4) have already been identified as key players in ferroptotic cell death, the involvement of nuclear events and epigenetic regulatory mechanisms remain largely unexplored. Only a handful of epigenetic enzymes and modifications, including KDM3B, LSH, and monoubiquitination of H2A-B, have been described as regulators of ferroptosis [22,23,24,25,26,27,28]. This is surprising since, similar to ferroptosis, the epigenetic machinery is heavily influenced by oxidative stress and the iron metabolism. Indeed, the activity of many epigenetic enzymes, including JmjC-domain-containing histone demethylases (JHDMs) and ten-eleven translocation (TET) DNA demethylases, is highly dependent on the availability of iron [29,30,31]. Similarly, hydroxyl radicals, produced during Fe^2+^-dependent Haber–Weiss reactions taking place in ferroptotic cells [32], can produce methyl radicals and cause non-enzymatic methylation of cytosine and guanidine residues in the DNA and directly impact methylome changes [33]. Finally, inhibition of cystine import by ferroptosis inducer erastin triggers the activation of the transsulfuration pathway [34], and might limit the availability of the methyl donors required for DNA and histone methylation [35,36]. Because epigenetic alterations often contribute to the development of therapy resistance and the malignant transformation of cancer cells, a better understanding of the interplay between ferroptotic cell death and epigenomic changes might further elucidate the therapeutic potential of ferroptotic compounds. In this study, we therefore combined RNA sequencing, LC-MS/MS, pyrosequencing, and EPIC BeadChip analysis to characterize the epigenetic changes taking place in therapy-resistant and -sensitive ferroptotic MM1 cells.

## 2. Results

### 2.1. Glucocorticoid-Sensitive and -Resistant MM1 Cells Exhibit a Similar Transcriptome Stress Response upon Ferroptotic Cell Death Induction

**Figure 1:**
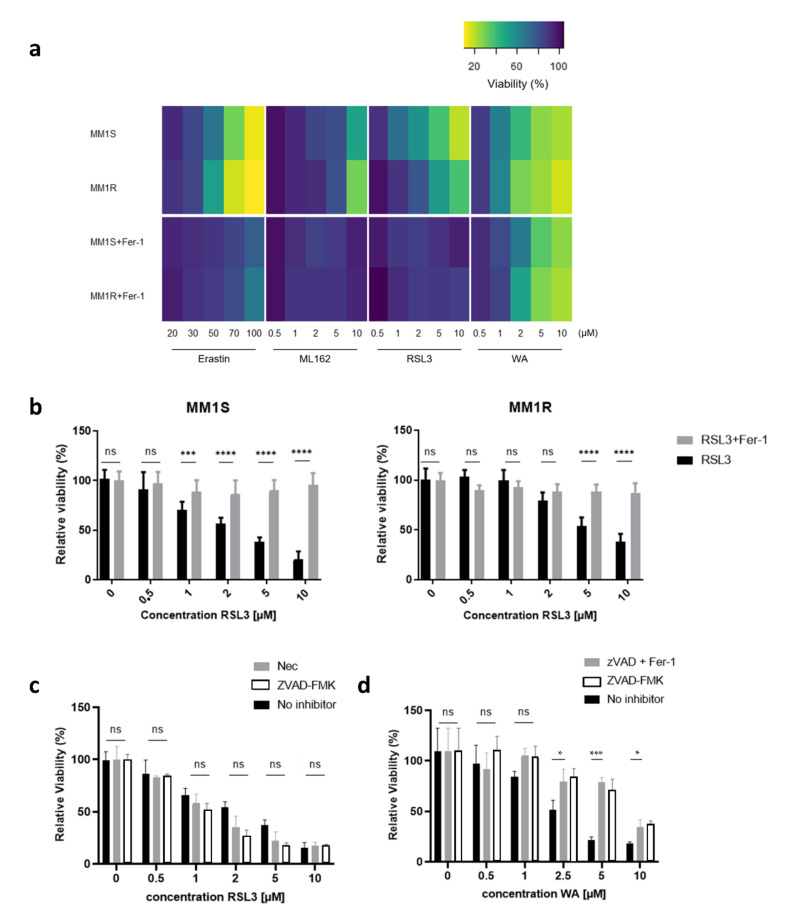
MM1 multiple myeloma cell lines are susceptible to ferroptosis induction. (**a**) Heatmap representing relative cell viability of MM1 cells upon 24 h exposure to increasing concentrations of erastin, ML162, RSL3, and withaferin A (WA) with or without pre-treatment with ferroptosis inhibitor ferrostatin-1 (Fer-1). (**b**) Relative cell viability of MM1S (left) and MM1R (right) cells upon 24 h exposure to increasing concentrations of RSL3 with or without pre-treatment with 2 µM ferrostatin-1 (Fer-1). Data are plotted as the mean ± s.d., *n* = 3 biologically independent replicates (ns = *p* > 0.05, *** *p* < 0.001 **** *p* < 0.0001, ANOVA). (**c**) Relative viability of MM1 cells upon 24 h exposure to increasing concentrations of RSL3 with or without pre-treatment with 10 µM necrostatin-1 (Nec) or 50 µM caspase inhibitor ZVAD-FMK. Data are plotted as the mean ± s.d., *n* = 3 biologically independent replicates. (**d**) Relative viability of MM1 cells upon 24 h exposure to increasing concentrations of WA with or without pre-treatment with 10 µM necrostatin-1 (Nec) or 50 µM caspase inhibitor ZVAD-FMK (* *p* < 0.05). Data are plotted as the mean ± s.d., *n* = 3 biologically independent replicates.

Acquired and primary therapy resistance remains a major hurdle in clinical treatment of MM. Despite the availability of broad-spectrum anti-cancer drugs, including glucocorticoids (GCs), proteasome inhibitors (PIs), and immunomodulatory drugs (IMiDs), most MM patients eventually relapse and become refractory to current treatments [37]. Therefore, we explored whether therapy-resistant MM cells are sensitive to ferroptosis induction, an iron-catalyzed mode of cell death associated with increased lipid peroxidation. In this study, we exposed GC-resistant (MM1R) and GC-sensitive (MM1S) MM1 cells to different ferroptosis inducers and evaluated their effect on cell viability (Figure 1a). Although both cell lines have the same origin, MM1R cells are known to be resistant to GC-mediated apoptosis due to their defective GC receptor (GR) and lack of GR expression. We observed that all compounds successfully induced cell death in both cell lines and that GPX4 inhibitor RSL3 was most potent, with IC50 values ranging between 2.82 µM and 6.31 µM (Figure 1b). RSL3-induced cell death could be fully prevented by pre-treatment with ferroptosis inhibitor ferrostatin-1 (Fer-1), but not by pre-treatment with necrostatin-1 (Nec) or ZVAD-FMK, which are necroptosis and apoptosis inhibitors, respectively (Figure 1c). Of note, pre-treatment with Fer-1 does not impact or reverse dexamethasone sensitivity of MM1 cells and indicates that other modes of RCD are involved in mediating GC-triggered cell death (Supplementary Figure S1). Similarly, only ZVAD-FMK was able to partially rescue cell death induced by Withaferin A (WA) (Figure 1d), a natural steroidal lactone that has previously been reported to display both apoptotic- and ferroptotic-mediated anti-cancer properties [38,39,40]. This suggests that in our MM cell model, WA promotes apoptotic rather than ferroptotic cell death. Indeed, although flow cytometry analysis revealed that RSL3-induced cell death was accompanied by an increase in lipid peroxidation after 3 h of treatment (Supplementary Figure S2a,b), this was not observed in cells incubated with WA (data not shown). The cell death modality induced by WA might therefore be highly dependent on cell type and context.

**Figure 2:**
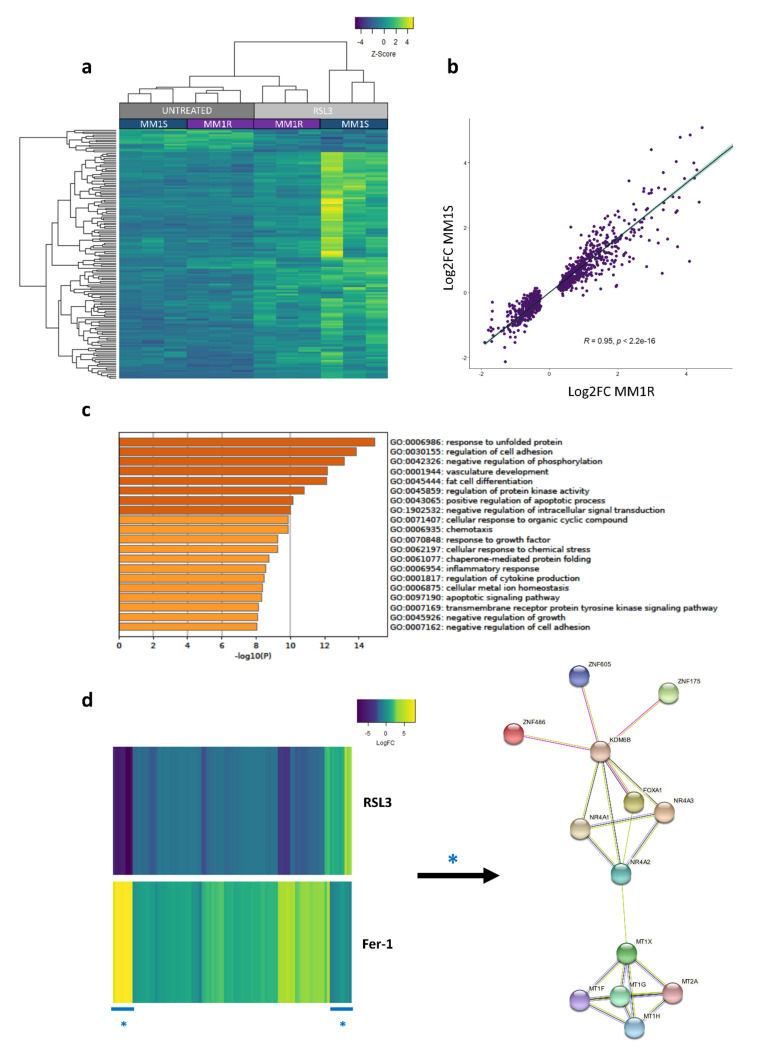
RNAseq analysis reveals RSL3-induced transcriptional changes in MM1 cells. (**a**) Heatmap representation of rlog gene counts of differentially expressed genes (FDR < 0.05, logFC > | 1 |) in MM1 cells treated with 5 µM RSL3 for 3 h. Gene counts are represented as Z-scores, *n* = 3 biologically independent replicates per cell line. (**b**) Scatter plot displaying the correlation between the logFC of significant DEGS identified in MM1R and MM1S cells. The strength of the correlation is indicated by the regression line (with 95% confidence interval indicated in light blue) and corresponding coefficient of determination (R). Significance of the correlation was calculated by Pearson correlation and is represented by the Pearson correlation coefficient (*p*). (**c**) Metascape pathway analysis [44] of RNAseq data displaying the top 20 significantly enriched pathways of RSL3-treated MM1 cells compared to untreated controls. (**d**) Heatmap representation (left) and protein interaction network (right) of differentially expressed genes in RSL3-treated MM1 cells, of which the expression change could be completely reverted by a 2 h 2 µM ferrostatin (Fer-1) pre-treatment. The most significant genes are indicated with * and included in the protein–protein interaction network.

To characterize transcriptional changes taking place in early ferroptotic MM1 cells, RNA sequencing analysis of RSL3-treated MM1 cells was performed and compared to untreated controls. Using selection criteria of FDR < 0.05 and logFC > | 1 |, changes in gene expression patterns in both MM1S and MM1R cells were shown to be highly similar (Figure 2a,b). A total of 616 common significant differentially expressed genes (DEGs) could be identified, the majority of which (540 DEGs) were found to be upregulated upon ferroptosis induction (including FTH1, FTL, CHAC1, HSPB1, SLC7A11, and HMOX [40,41,42,43]) (Supplementary Figure S3a,b)). Metascape pathway analysis of DEGs revealed an enrichment in inflammation, kinase signaling, cellular stress, and cell death-related pathways (Figure 2c). Remarkably, altered expression of 95 of the 616 identified DEGs could completely be reverted by a 2 h pre-treatment with 2 µM Fer-1 (FDR < 0.05, logFC | 1 |), suggesting that these genes in particular are crucial for ferroptosis induction (Figure 2d). The most significant genes within this subset are involved in metal binding (including metallothioneins [MT1s] and zinc finger proteins [ZNF]), nuclear receptor signaling (orphan nuclear receptor 1–3 (NR4A1-3)), chromatin remodeling (NR4A2, FOXA1, KDM6B), and gene transcription (ZNFs, NR4A1-3) (Figure 2d). Taken together, these findings suggest that ferroptosis effectively targets MM1 cells and that RSL3-mediated ferroptosis triggers similar oxidative stress and cell death pathways in both MM1R and MM1S cells, irrespective of their GC-sensitivity status. Moreover, genes involved in metal binding and chromatin remodeling seem to be crucial for mediating ferroptotic cell death.

### 2.2. RSL3-Mediated GPX4 Inhibition Triggers the Formation of Non-Heme Iron-Histone Complexes and DNA Damage Responses

Because RNAseq data revealed the involvement of chromatin remodelers, including FOXA1, NR4A2, and KDM6B, in ferroptotic cell death, we next utilized a LC-MS/MS approach to investigate whether RSL3-induced oxidative stress signals propagate to the nucleus and alter histone post-translational modifications (Supplementary Figure S4a) [45]. First, LC-MS-compatible cell lysis and histone extraction protocols were optimized to obtain pure histone extracts (Supplementary Figure S4b) [46]. Overall, direct acid extraction of histones from whole cell pellets resulted in higher protein yield compared to hypotonic lysis where nuclei are isolated prior to acid extraction (Supplementary Figure S4c).

**Figure 3:**
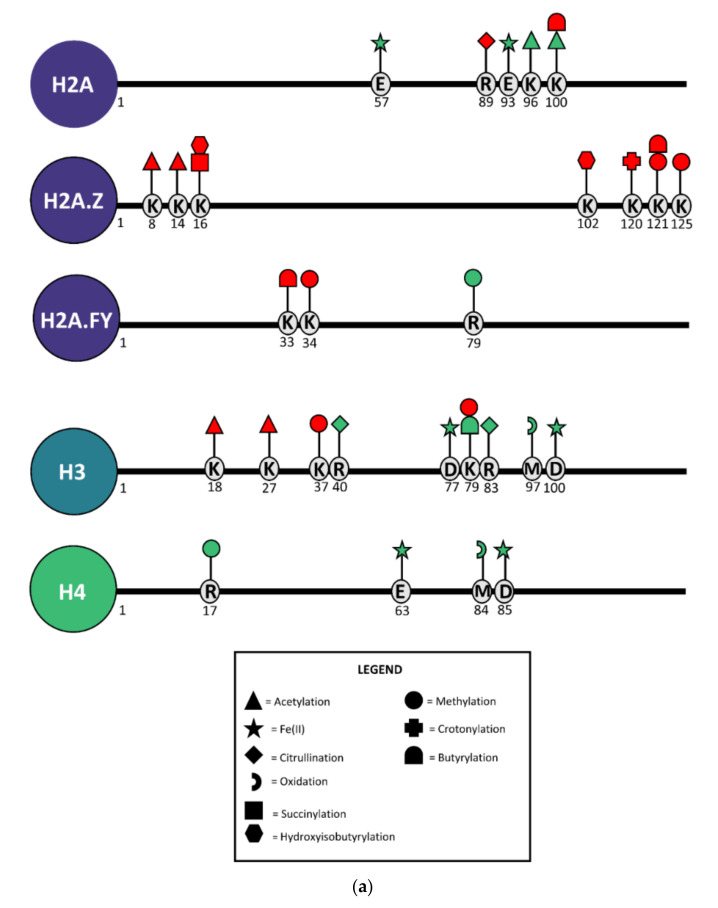

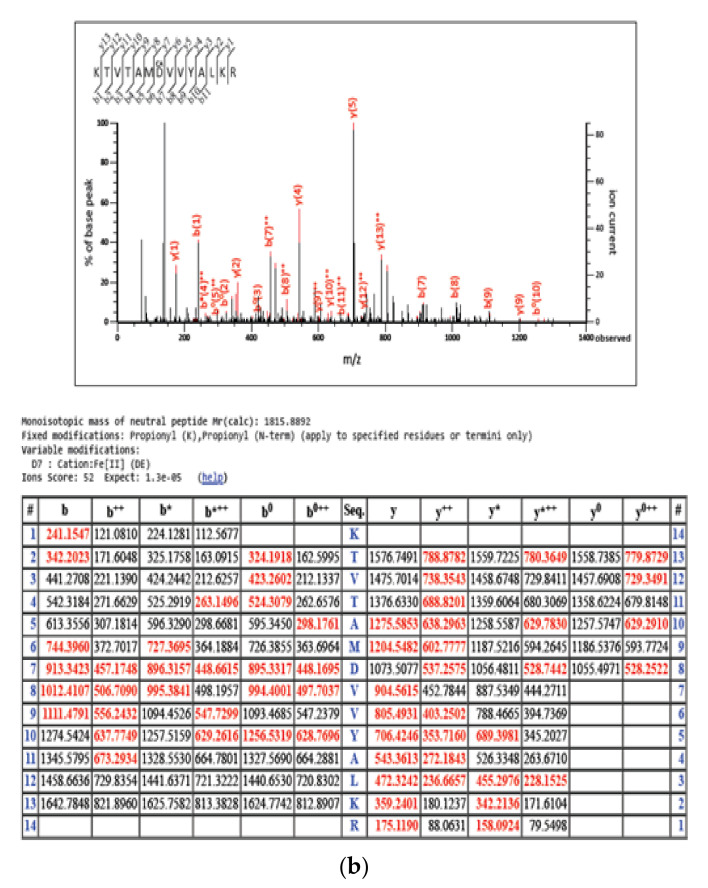
(**a**) Overview of progressive significant (*p* < 0.05) changes in histone post-transcriptional modifications (PTMs) in MM1 cells after RSL3 treatment compared to untreated controls. Each symbol depicts a different PTM. Red symbols represent downregulated modifications whereas green symbols represent upregulation. The position of each PTM is indicated by the amino acid (AA) single-letter code and its AA position within the corresponding histone protein sequence. (**b**) Annotated H4 MS/MS spectrum demonstrating Fe^2+^ binding to H4 glutamate (E) and aspartate (D) residues under ferroptotic conditions. Coverage of detected b and y ions are presented in the lower table, with # indicating fragment index and “Seq.” referring to the amino acid residue. B and y ions displayed in the figure are singly charged, unless otherwise indicated by their index (++ indicates a doubly charged fragment ions). Ions marked with an asterisk (*) represent ions with ammonium loss, ions marked with a circle (°) represent ions with water loss.

After optimization, histone epigenetic profiling was performed using an untargeted, data-dependent acquisition (DDA) MS-based screening method [45]. Briefly, MM1 cells were treated with 5 µM RSL3 for increasing time periods (1h, 2h, 4h, 8h), after which histones were extracted, propionylated, and trypsinized. Next, MS1 and MS2 spectra were obtained by HPLC-MS/MS analysis and raw data were analyzed with the Progenesis QIP 3.0 software (Waters). Ultimately, relative abundances of histone PTMs were obtained and statistical analysis was performed to identify significantly altered PTMs (Supplementary Table S1). Overall, we observed that cells treated with RSL3 for increasing time periods showed a progressive (i.e., continuous change observed in all included treatment timepoints) increase and decrease in several histone PTMs (Figure 3). In line with our RNAseq data, where most genes were found to be upregulated upon RSL3 induction, ferroptotic cells portrayed a genome-wide, time-dependent decrease in KDM6B-mediated repressive histone mark H3K37me3 [47]. However, loss of active marks, including H3K18ac, H3K79me3, and H3K27ac, was also detected upon continued RSL3 treatment (Supplementary Figure S5a).

Remarkably, H3K79me3, H3K18ac, and H3K27ac have all been associated with DNA damage, cellular senescence, and genome stability (Table 1), suggesting that RSL3-mediated oxidative stress might drive these processes in MM1 cells. This is also reflected by the increased detection of histone variants H2A.Z and H2A.FY (macroH2A1), which are known to regulate DNA repair and cellular senescence (Table 1). Moreover, Western blot analysis revealed that prolonged exposure (>12 h) to RSL3 resulted in a significant increase in pH2AX, a key maker for double-stranded DNA damage (Supplementary Figure S5b).

Next to histone PTMs associated with DNA damage and cellular senescence, we found a significant increase in histone acylation modifications, including succinylation, crotonylation, and (hydroxyiso)butyrylation (Figure 3, Supplementary Table S1). Although the functional effects of these PTMs are far less characterized compared to those of histone acetylations, several studies highlight that they are heavily influenced by fatty acid metabolism (reviewed in [73]). Depending on the cellular metabolic state, crucial metabolic intermediates (e.g., crotonate) are increased or decreased, impacting their availability for acyl-transferase-mediated histone modifications. Given that ferroptosis is hallmarked by lipid peroxidation and overall changes in the lipid metabolism [74], these histone acylation changes might directly reflect the altered metabolic state of ferroptotic cells and regulate gene expression accordingly [75,76,77].

**Figure 4:**
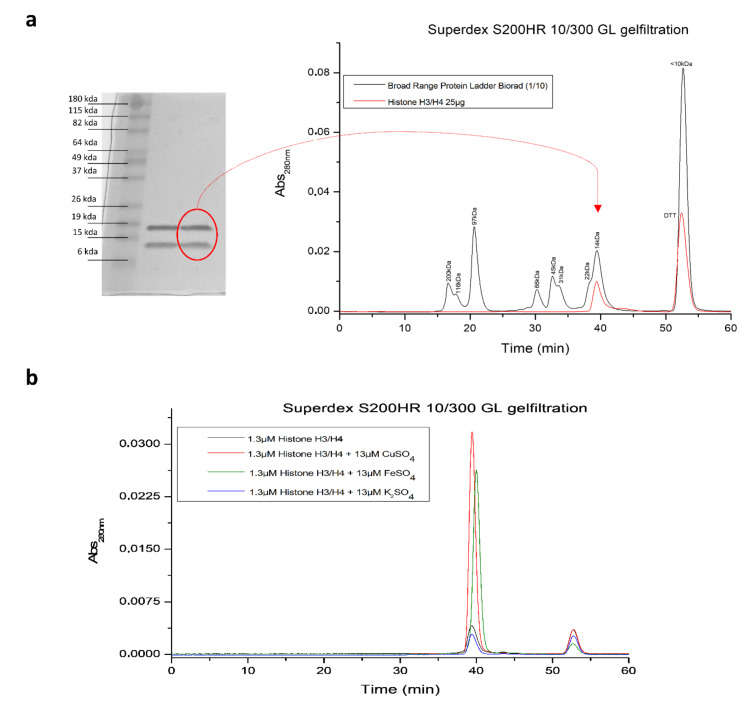
Purified H3-H4 tetramers display an increase in 280 nm absorbance after addition of FeSO_4_. (**a**) Purity of commercial H3-H4 tetramers was evaluated by SDS-PAGE Coomassie staining (left) and gel filtration (right). (**b**) Absorbance spectrum at 280 nm of H3-H4 tetramers incubated with a 10-fold excess of CuSO_4_ (positive control), K_2_SO_4_ (negative control), and FeSO_4_.

Surprisingly, through an error-tolerant search strategy, we discovered that the altered iron metabolism during ferroptosis directly affects histone PTMs as well. Specifically, several glutamate (E) and aspartate (D) residues in H2A, H3, and H4 histone tails were significantly enriched in Fe^2+^ binding, a modification that we have never seen before in over 5000 LC-MS runs acquired on histones at the lab (Figure 3a,b, Supplementary Figure S6, Supplementary Table S1). To the best of our knowledge, we are the first research group to describe Fe^2+^ binding to histone proteins. The biological relevance of the formation of non-heme iron histone complexes needs to be further explored, but might include a role in iron chelation in response to the ferroptosis-driven intracellular increases in labile iron, regulation of gene expression, DNA damage, and DNA damage protection, or even reflect the oxidoreductase activity of H3-H4 tetramers as previously reported for Cu^2+^ [78]. To further investigate the iron-binding properties of histone proteins, commercial H3-H4 tetramers were incubated with FeSO4 and separated by gel filtration, after which absorbance at 280 nm was measured. Similar to the absorbance signal measured in the CuSO4-positive control, H3-H4 tetramers incubated with FeSO4 demonstrated an increased absorbance at 280 nm (Figure 4). Furthermore, incubated FeSO4 resulted in a small shift in H3-H4 protein size, suggesting that Fe^2+^ binding affects the structural organization of the tetramer complex. Overall, our data suggest that histone proteins and PTMs are sensitive to altered oxidative stress, as well as fatty acid and iron metabolism changes mediated by GPX4 inhibition.

### 2.3. Prolonged Exposure of MM1 Cells to RSL3 Results in Local DNA Methylation Changes and Promotes Expression of DNA Damage Repair Protein NR4A2

**Figure 5:**
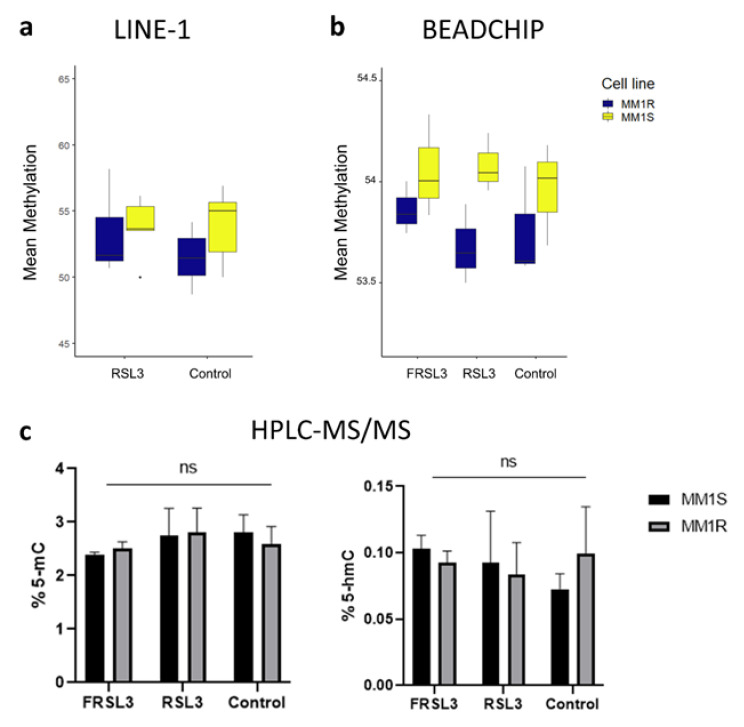
Global DNA methylation changes in MM1 cells after RSL3 treatment (with or without pre-treatment of ferrostatin-1 (FRSL3)). (**a**) Mean methylation levels (%) of 3 CpGs located in long interspersed nuclear elements 1 (LINE-1), *n* = 3 biologically independent replicates. (**b**) Mean methylation levels (%) of 866.836 genome-wide CpG sites analyzed with the Illumina Infinium Methylation EPIC BeadChip, *n* = 3 biologically independent replicates. (**c**) Mean % of 5-methylcytosine (left) and 5-hydorxymethylcytosine (right) residues present in MM1 samples. Data are represented as the mean ± s.d. (ns = *p* > 0.05, ANOVA), *n* = 3 biologically independent replicates.

Taking into account that ferroptotic stress directly affects expression of chromatin remodelers and histone PTMs, we wondered whether DNA methylation changes also occur upon RSL3 induction in MM1 cells. Given that prior studies have reported significant global DNA methylation alterations upon intracellular increases in reactive oxygen species (ROS) and iron [33,79,80], we first evaluated changes in global DNA methylation levels in MM1S and MM1R cells treated with 1 µM RSL3 for 72 h. In contrast to prior described experiments (Section 2.1 and Section 2.2), cells were treated with subtoxic concentrations of RSL3 (1 µM) to maintain high cell viability upon prolonged (72 h) RSL3 treatment. These prolonged treatment times ensure that de novo DNA methylation changes are also included in the analyses (doubling times of MM1 cells is 72 h). Three independent methods, namely, LINE-1 pyrosequencing, Infinium Methylation EPIC BeadChip analysis, and HPLC-MS/MS analysis, revealed no significant shifts in DNA (hydroxy)methylation after chronic GPX4 inhibition (Figure 5a–c). All methods did show, however, that basal DNA methylation levels in MM1S cells were consistently higher compared to MM1R cells (Figure 5a–c).

**Figure 6:**
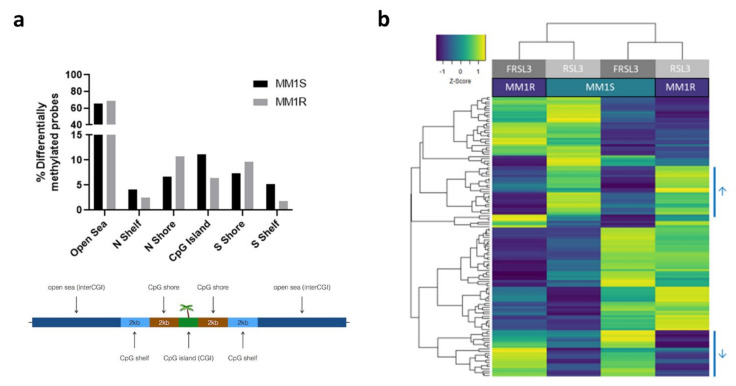
Local DNA methylation changes in MM1 cells after RSL3 treatment (with or without pre-treatment with ferrostatin-1 (FRSL3)). (**a**) Bar chart visualization of the percentage of significantly differentially methylated (FDR < 0.05, Δβ difference > 0.10) CpG loci (y-axis) between RSL3 and ferrostatin-1 pre-treated RSL3 MM1 cells and their corresponding genomic compartments (x-axis). (**b**) Heatmap representation of the methylation level (β-values) of the top common DMP CpG sites of MM1 cells treated with RSL3 (with or without pre-treatment with ferrostatin-1). β-values are represented as Z-scores, where the lowest methylation value is indicated in blue and the highest in yellow.

Next, local changes in DNA methylation of CpG sites were explored through Infinium Methylation EPIC BeadChip analysis, which measures mean methylation levels of 866,836 genome-wide CpG sites. CpG loci of RSL3-treated cells were compared to cells treated with Fer-1 and RSL3 and were considered to be differentially methylated if a difference of ≥10% methylation and FDR < 0.05 could be detected. Overall, RSL3 treatment resulted in a large number of differentially methylated probes (DMPs) in both MM1R (51 168 CpG probes corresponding to 487 genes) and MM1S cells (32 813 CpG probes corresponding to 408 genes). The majority of DMPs in both cell lines were located in open sea regions located > 4 kb from CpG islands (Figure 6a). Remarkably, when comparing the top significant probes (FDR < 0.01) between both cell lines, the beta values of ferroptotic MM1R cells clustered with the beta values of Fer-1-treated MM1S cells and ferroptotic MM1S cells clustered with Fer-1-treated MM1R cells, suggesting that both cell lines converge towards a similar methylation profile under ferroptotic conditions (Figure 6b). In line with this observation, genes associated with the significant DMPs (FDR < 0.05 and beta value > 0.1) were distinct in both cell lines but were enriched in several common pathways, including cell cycle, autophagy, and insulin signaling (Table 2, Supplementary Figures S7 and S8).

**Figure 7:**
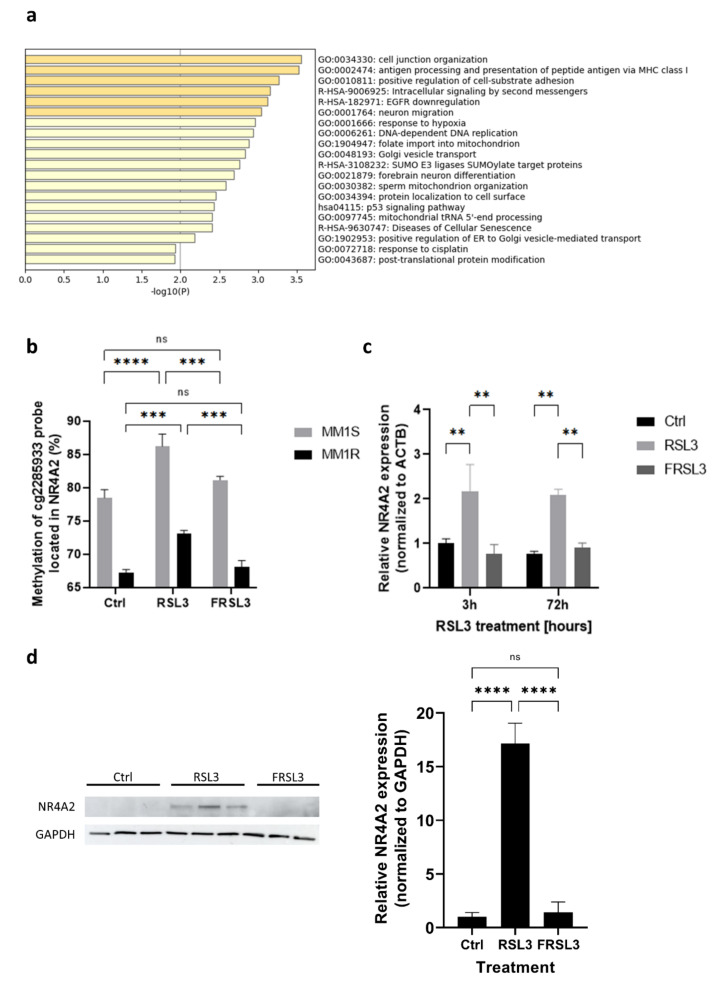
Ferroptosis induction results in significant methylation changes in genes associated with DNA repair and cellular senescence. (**a**) Metascape pathway analysis [44] of genes associated with the top significant (FDR < 0.01) differentially methylated CpG probes in MM1 cells after RSL3 treatment. (**b**) Mean methylation levels (%) of CpG probe cg2285933 located in the S shelf of the NR4A2 gene as determined by pyrosequencing. Data are represented as mean methylation levels ± s.d., *n* = 3 biologically independent replicates per cell line (*** *p* < 0.001, **** *p* < 0.0001). (**c**) Relative NR4A2 mRNA expression in MM1 cells after treatment with RSL3. Data are plotted as the mean ± s.d., *n* = 3 biologically independent replicates (** *p* < 0.01). (**d**) Western blot detection and quantification of NR4A2 and GAPDH expression levels after RSL3 treatment in MM1 cells. Data are plotted as the mean ± s.d., *n* = 3 biologically independent replicates per cell line (ns = *p* > 0.05, **** *p* < 0.0001, ANOVA).

Ferroptosis induction by RSL3 also resulted in specific hyper- and hypomethylation of a subset of probes in both cell lines (indicated by arrowheads in Figure 6b). Genes associated with these probes were mainly enriched in cell adhesion, inflammation, DNA replication, mitochondrial organization, cell death, and cellular senescence (Figure 7a). Of note, one of these genes included NR4A2, which we previously identified as a top significant DEG in our RNAseq data (see Section 2.1.). Both Infinium Methylation EPIC BeadChip and pyrosequencing analysis demonstrated that one of the probes associated with NR4A2 (cg2285933—located in the S shelf region of NR4A2) was significantly hypermethylated upon RSL3 treatment (Figure 7b) and that this hypermethylation was linked with increased mRNA and protein expression (Figure 7c,d). Similar observations have previously been made for another nuclear receptor gene, NR3C1, where hypermethylation outside of the CpG island led to an increase in gene expression [81].

## 3. Discussion

The majority of available anti-myeloma drugs, including GCs, PIs, and IMiDs, aim to eradicate tumor cells through induction of apoptosis [82]. However, most myeloma cells acquire resistance to this mode of cell death by upregulating anti-apoptotic proteins and downregulating pro-apoptotic signals, which is often driven by (epi)mutational changes [83,84]. For that reason, we explored whether the induction of ferroptotic cell death might be an effective alternative to eliminate GC-resistant myeloma cells. Our data show that GPX4 inhibitor RSL3 successfully targets MM1 cells, irrespective of their GC sensitivity status, and that induction of ferroptosis is linked to a common transcriptional response. Specifically, ferroptotic myeloma cells upregulate a significant number of genes involved in cellular stress and cell death pathways, inflammation, and fatty acid metabolism. Among these genes, chromatin remodelers, such as NR4A2, FOXA1, and KDM6B, seem to be pivotal in triggering ferroptosis. To this end, we further explored the epigenetic changes in ferroptotic myeloma cells and found that ferroptosis is associated with significant changes in several histone modifications and variants, most of which have previously been linked with DNA damage and cellular senescence pathways. Interestingly, RSL3 treatment also caused an increased binding of Fe^2+^ ions to glutamate and aspartate residues of H2, H3, and H4, suggesting that the ferroptosis-driven changes in intracellular iron concentrations are propagated to the nucleus. The formation of non-heme iron-histone complexes could be confirmed in gel filtration chromatography experiments, where H3-H4 tetramers showed an increased absorption upon FeSO_4_ incubation. However, further validation in other cell models and with other biochemical techniques, including isothermal titration calorimetry, circular dichroism spectrometry, or microscale thermophoresis, is required to determine whether this increased Fe^2+^ binding to histone proteins is a universal phenomenon in ferroptotic cells. If this is the case, the biological relevance of iron-histone complexes needs to be explored as well. Possibly, histone proteins mediate a genoprotective role by chelating iron and preventing DNA damage [85]. Because earlier experiments revealed a cupper binding-dependent reductase activity of H3-H4 tetramer complexes [78], Fe^2+^ binding to histones might also fuel an enzymatic function of histone proteins that has yet to be uncovered. Alternatively, histones are known to function as oxygen and nutrient sensors and the observed iron-binding might be a reflection of this role [86,87]. In line with this latter hypothesis, significant changes in different histone acylation marks, which are known to respond to metabolic perturbations [88], were detected in ferroptotic myeloma cells as well.

Since alterations in oxidative stress and intracellular iron concentration are known to directly impact DNA methylation [31,33,79,80], we next investigated whether ferroptosis impacts DNA methylation levels in MM1 myeloma cells. Although ten-eleven translocation (TET) DNA demethylation enzymes are known to be sensitive to changes in labile iron [89,90], no significant changes in global DNA (hydroxy)methylation in RSL3-treated cells could be observed by LINE-1 pyrosequencing, HPLC-MS/MS analysis, or BeadChip EPIC methylation arrays, indicating that the overall activity of these TET enzymes is not altered during ferroptosis. In contrast, BeadChip EPIC methylation analysis did uncover local DNA methylation changes of numerous DMPs in both GC-sensitive MM1S and GC-resistant MM1R ferroptotic cells. Clustering analysis of the top significant DMPs (FDR < 0.01) revealed two major trends in the methylation data. First, RSL3-mediated GPX4 inhibition drives MM1S and MM1R cells towards a similar methylation profile, indicating that ferroptosis triggers a common stress epigenome response in cells with different epigenetic backgrounds. This implies that ferroptosis signaling either differentially methylates DMPs located in MM1S but not in MM1R (or vice versa) or that both cell lines undergo methylation changes in opposite directionalities (i.e., hypermethylated in MM1R, hypomethylated in MM1S). Nevertheless, both cell lines portray similar RSL3-dependent transcriptional changes and sensitivities to ferroptotic compounds, suggesting that most of these DNA methylation changes are epigenetically redundant. Second, similar to the histone proteomics data, ferroptotic cell death induction resulted in specific hyper- and hypomethylation of genes involved in DNA repair, cellular senescence, and cell death pathways. Of note, NR4A2 expression was found to be upregulated upon methylation of its S shore region. NR4A2, also known as Nurr1, is involved in mediating DNA damage repair responses induced by genotoxic triggers [91,92] and has been identified as a potential target for anti-aging interventions (reviewed in [93]).

Although we did not directly assess expression of senescent markers of ferroptotic myeloma cells in this study, both our histone proteomics and DNA methylation data suggest cellular senescence pathways are altered during ferroptosis. Generally, cellular senescence is described as a state of permanent growth arrest that can be induced by cellular stress or DNA damage [94]. Western blot analysis of pH2AX, a marker of double-stranded DNA breaks, indeed shows an increase in DNA damage upon prolonged RSL3 stimulation in MM1 cells. Other proteins involved in the DNA damage response (DDR), including p53 and ATM/ATR kinases, have recently also been linked to ferroptotic cell death [95]. In agreement with our observations, studies in neuronal and retina epithelial cells have revealed that ferroptotic compounds are able to promote cellular senescence [96,97,98]. Whether cellular senescence promotes sensitization [97,99] or resistance [100] to ferroptosis is currently still under debate. The ferroptosis-driven epigenetic changes characterized in this study seem to suggest that RSL3-mediated oxidative stress orchestrates DNA damage and genomic instability, which induces premature cell senescence and prompts activation of DNA repair genes such as NR4A2. Interestingly, prior research reported that drug-induced senescence promoted MM cell recognition by natural killer cells and elimination of tumor cells [101]. Similarly, ferroptosis-mediated senescence might provide new insights for the exploitation of senolytic drugs in cancer therapies that specifically target senescent cells [102].

Nevertheless, it remains challenging to assess whether the identified ferroptosis-associated epigenetic alterations are a cause or consequence of ferroptosis induction. On the one hand, ferroptosis signaling is known to indirectly change the activity of multiple epigenetic enzymes by influencing the iron-redox metabolism [103,104], but on the other hand, studies with epigenetic drugs observed significant changes in ferroptosis therapy sensitivity [27,105]. To this end, functional assays, animal studies, and longitudinal studies should be applied to independently prove the causality and functionality of specific epigenetic marks in ferroptotic cell death. Of particular interest, novel CRISPR/Cas9-based epigenetic editing techniques could help to investigate the causative relationship between ferroptosis induction and some of the epigenetic marks [106]. When these epigenetic marks prove to be non-causal, they could alternatively be used as predictive or prognostic markers for ferroptosis sensitivity, as ferroptosis resistance has already been described in some cancer types [40,107]. Given that cancer-therapy resistance is often orchestrated by epigenetic alterations, an epigenome comparison of ferroptosis resistance and sensitivity might help to identify novel ferroptosis biomarkers and predict efficacy of ferroptosis inducers.

## 4. Materials and Methods

### 4.1. Antibodies and Reagents

Ferroptosis inducers erastin (S7242) and RSL3 (S8155), and cell death inhibitors ferrostatin-1 (S7243) and necrostatin (S8037), were purchased from Selleckchem (Houston, TX, USA). ML162 (SML2561) and ZVAD-FMK (V166) were purchased from Sigma-Aldrich (Saint Louis, MO, USA). WA was obtained from Alta Vista Phytochemicals (Hyderabad, India). All compounds were dissolved in DMSO at a stock concentration of 20 or 50 mM. 

Primary antibodies targeting pH2AX (ab81299), H3K27ac (ab4729), H3K36me3 (ab9050), and GAPDH (ab9485) were obtained from abcam (Cambridge, UK). The NR4A2 antibody (Nurr1 monoclonal antibody, MA1-195) was purchased from ThermoFisher Scientific (Waltham, MA, USA).

### 4.2. Cell Culture and Cell Viability Assays

MM1R and MM1S cells were cultured in RPMI 1640 medium supplemented with 10% fetal bovine serum (E.U. Approved; South American Origin), 1% MEM non-essential amino acids (Invitrogen, Carlsbad, CA, USA), 1% sodium pyruvate (Invitrogen, Carlsbad, CA, USA), and 1% penicillin/streptomycin solution (Invitrogen, Carlsbad, CA, USA). Cell viability was assessed by the MTT colorimetric assay (Sigma Aldrich, St. Louis, MO, USA) as previously described [108].

### 4.3. Lipid Peroxidation Assay

Cellular lipid reactive oxygen species were measured using the Image-iT™ Lipid Peroxidation Kit (C10445, ThermoFisher Scientific, Waltham, MA, USA) according to the manufacturer’s protocol. In short, cells were seeded in 6 well plates at a density of 5 × 105 cells/well and treated the next day with 5 µM RSL3 (with or without pre-treatment with 2 µM Fer-1) or 100 µM cumene hydroperoxide (positive control). Cells were subsequently incubated for 30 min with 10 µM Image-iT™ Lipid Peroxidation Sensor at 37 °C. After incubation, cells were collected by trypsinization with TrypLE Express Enzyme (ThermoFisher Scientific, Waltham, MA, USA). Cells were washed 3 times with pre-warmed PBS and a fluorescence shift from 590 nm to 510 nm was measured with the CytoFlex flow cytometer (Beckman Coulter Life Sciences, Indianapolis, IN, USA).

### 4.4. RNA Extraction, RNA Sequencing, and RNA Sequencing Data Analysis

Total RNA from control and RSL3-treated (3 h, 5 µM RSL3) MM1 cells was isolated using the RNeasy Mini Kit (Qiagen, Venlo, Netherlands) according to the manufacturer’s protocol. Isolated RNA was quantified and qualified using the Epoch™ Microplate Spectrophotometer (BioTek, Winooski, VT, USA) and sent to BGI (BGI Group, Beijing, China) for RNA sequencing analysis as previously described [108]. Briefly, RNA integrity (RNA content > 80 ng/µL, 28 s/18 s ≥ 1.0 and RIN ≥ 7.0) was determined using the 2100 Bioanalyzer system (Agilent Technologies, Santa Clara, CA, USA), after which library preparation was initiated. A 2 X 50 bp pair-end RNA sequencing was subsequently performed on the BGISEQ-500 platform (BGI Group, Beijing, China). RNAseq data were deposited in the NCBI GEO database with accession number GSE22309570.

Analysis of RNA sequencing data was performed as previously described [108]. In short, the quality of the sequencing reads was evaluated using FastQC (v0.11.5, Braham Institute, Cambridge, UK) [109] and subsequent alignment to the human reference genome build 37 (hg19) was performed with the STAR (v.2.7.3a, Cold Spring Harbor Laboratory, Cold Spring Harbor, NY, USA) tool [110]. Differential gene expression was performed with the DESeq2 R package (v1.30.1, European Molecular Biology Laboratory, Heidelberg, Germany) using the Wald statistical test, BH-corrected for multiple testing [111]. Genes were considered statistically significant when FDR < 0.5. Metascape pathway analysis of the statistically significant DEGs was performed with the online Metascape web tool (v3.5, Genomics Institute of the Novartis Research Foundation, San Diego, CA, USA) using the express analysis settings [44]. Pearson’s correlation of differentially expressed genes between MM1S and MM1R cells was visualized using the ggscatter function of the gplots R package (v3.1.1, Tel Aviv University, Tel Aviv, Israel) [112]. Protein interaction networks were generated using the STRING database (v11—medium confidence interaction score settings, University of Zurich, Zurich, Switzerland) [113].

### 4.5. cDNA Synthesis and qPCR Analysis

Total extracted RNA (500 ng) was converted into cDNA using the GoScript™ Reverse Transcriptase System (Promega, Madison, WI, USA) according to the manufacturer’s instructions. cDNA was subsequently used as input for qPCR analysis using the GoTaq^®^ qPCR Master Mix (Promega, Madison, WI, USA) as previously described [108]. ΔΔCt-values were calculated using ACTB as a housekeeping gene. Primers sequences are listed in Supplementary Table S2.

### 4.6. Histone Extraction and MS Sample Preparation

Extraction of histone proteins was performed as previously described [45,46]. In short, two extraction protocols, namely, hypotonic lysis (protocol A) and direct acid lysis (protocol B), were explored. In protocol A, 2 × 10^6^ cells were resuspended in hypotonic lysis buffer (10 mM Tris-HCl pH 8.0, 1 mM KCl, 1.5 mM MgCl2) supplemented with 1 mM DTT, 1 mM PMSF, Halt Protease and Phosphatase Inhibitor Cocktail 100× (ThermoFisher Scientific, Waltham, MA, USA), and phosphatase inhibitor cocktails II and III (Sigma-Aldrich, Saint-Louis, MO, USA). Lysates were incubated for 30 min at 4 °C on a rotator, after which nuclei were pelleted by 10 min centrifugation at 10,000× *g* and 4 °C. Pellets were then resuspended in 0.4 N HCl and incubated again at 4 °C for 30 min on a rotator. In protocol B, 2 × 10^6^ cells were lysed directly in 0.4 N HCl and incubated at 4 °C on a rotator for 2 h. For both protocols, lysates were subsequently centrifuged (16,000× *g*, 10 min, 4 °C) and supernatant was transferred to new Protein LoBind Eppendorf tubes. A final concentration of 33% trichloroacetic acid was slowly added to the histone solution, after which samples were inverted several times and incubated on ice for 30 min. Pelleted (16,000× *g*, 10 min, 4 °C) histones were washed twice with ice-cold acetone and air dried in a fume hood at RT. Part of the extracted histones (corresponding to 4 × 10^5^ cells) were used to assess purity and quantity by gel electrophoresis. Dried histones were resuspended in 2× Laemmli buffer (2.14% SDS, 26.3 % glycerol, and 10% 2-mercaptoethanol in 65.8 mM Tris-HCl pH 6.8) and separated on 8–16% Criterion TGX™ gradient gels (Bio-Rad Laboratories, Hercules, CA, USA). Gels were stained with the Sypro Ruby fluorescent gel stain (ThermoFisher Scientific, Waltham, MA, USA) and visualized with the VersaDoc 3000 imager (Bio-Rad Laboratories, Hercules, CA, USA). Quantity One software (Bio-Rad Laboratories, Hercules, CA, USA) was used for purity analysis. Remaining purified histones were propionylated and trypsin digested as previously described [46]. Proprionylated histones were dissolved in 0.1% formic acid in UPLC-grade water, sonicated, and centrifuged to remove insoluble aggregates prior to injection onto the LC-MS system. Equal fractions of all samples were pooled to generate quality control (QC) samples, which were run in fixed intervals in between the other samples. All samples were spiked with digested beta-galactosidase (20 fmol on column; Sciex) to monitor chromatographic quality and variation between LC-MS runs.

### 4.7. LC-MS Method and Data Analysis

All samples were acquired in data-dependent acquisition (DDA) using a TripleTOF 5600 mass spectrometer (Sciex, Concord, Ontario, Canada) coupled to a NanoLC 400 HPLC system (Eksigent, Dublin, CA). Histone samples were loaded onto a YMC TriArt C18 trap column (id 500 µm, length 5 mm, particle size 3 µm) at a flow rate of 5 μL/min for 5 min in 0.1% trifluoroacetic acid (TFA) in water. Afterwards, histone peptides were transferred to a microLC YMC TriArt C18 column (id 300 µm, length 15 cm, particle size 3 µm) and separated at a flow rate of 5 µL/min using a gradient of 60 min going from 3 to 45% mobile phase B. Mobile phase A consisted of UPLC-grade water spiked with 0.1% (*v/v*) FA and 3% (*v/v*) DMSO, whereas mobile phase B consisted of UPLC-grade ACN spiked with 0.1% (*v/v*) FA. The 10 most intense precursor ions from 400–1250 m/z with charge states 2–5 that exceeded 300 counts per second were selected for fragmentation, and the corresponding fragmentation MS2 spectra were collected between 65–2000 m/z for 200 ms. After the fragmentation event, the precursor ions were dynamically excluded from reselection for 10 s. Progenesis QI for Proteomics (Progenesis QIP v4.1, Nonlinear Dynamics, Waters) was used to process the raw LC–MS data as described before [46]. In short, raw data were imported and aligned in the Progenesis software, after which the detected peptide spectra were annotated to histone PTMs using Mascot (Matrix Science) with the same settings as described in [44]. Once histone PTMs were identified, relative abundances of each PTM were calculated by dividing the area under the curve (AUC) of each peptidoform containing the PTM by the sum of AUCs for all observed forms of that peptide. Significant (*p* < 0.05) differences in PTM relative abundance between treatment groups and the untreated controls were identified by performing two-tailed student *t*-tests.

### 4.8. Protein Extraction and Western Blot Analysis

To validate and quantify histone PTMs, 6 µg of purified histone extracts were resuspended in 4 X Laemmli buffer diluted in high-purity water. Protein separation and Western blot detection were performed as previously described [108]. Whole-cell lysates of RSL3-treated cells were obtained by lysing cell pellets (1 × 10^6^ cells) in 0.5 mL RIPA buffer (150 mM NaCl, 0.1% Triton X-100, 0.1% SDS, 50 mM Tris-HCl pH 8) supplemented with protease inhibitors (Complete Mini^®^, Roche). Soluble protein extracts were obtained after 15 min incubation on ice followed by brief sonication and centrifugation at 16 g for 20 min at 4 °C. All blots were blocked for 1 h at RT in 5% BSA blocking buffer and incubated overnight with primary antibody at 4 °C. After 2 h incubation at RT with dye-conjugated secondary antibody (Dako, Glostrup, Denmark), signal intensities were measured with the Amersham Imager 680 (Cytiva, MA, USA) and quantified with Image J software.

### 4.9. Gel Filtration Chromatography

Commercially available H3-H4 tetramers (Epicypher, Durham, NC, USA) were incubated for 30 min with a tenfold excess of FeSO_4_, CuSO_4_ (positive control), or K_2_SO_4_ (negative control). After 15 min centrifugation at 13,400 rpm, samples were loaded on a Superdex 200 HR 10/300 GL gel filtration column (Cytiva, MA, USA) equilibrated with running buffer (500 mM NaCl, pH 7.2) at a flow rate of 0.5 mL/min. Absorbance was measured at 280 nm and retention times were compared to those of the Biorad Broad Range Standard (Biorad, CA, USA).

### 4.10. DNA Extraction and Bisulfite Conversion

Total DNA from MM1 cells was isolated using the QIAmp DNA mini kit (Qiagen, Venlo, Netherlands) according to the manufacturer’s instructions. Subsequent bisulfite conversion of 1 µg isolated DNA was performed using the EpiTect Fast Bisulfite Conversion kit (Qiagen, Venlo, Netherlands) and the EZ DNA Methylation Kit (Zymo Research, Irvine, CA, USA) for pyrosequencing and Infinium MethylationEPIC analysis, respectively. To confirm successful bisulfite conversion, a PCR using bisulfite-specific primers was executed with the PyroMark PCR kit (Qiagen, Venlo, Netherlands) and the resulting PCR product was visualized on a 2% agarose gel to which GelRed™ staining (Biotium, Fremont, CA, USA) was added. Primers sequences are listed in Supplementary Table S2.

### 4.11. LINE-1 Pyrosequencing

Methylation of LINE-1 in MM1 cells was analyzed by pyrosequencing, as previously described [114]. Briefly, all required primers (i.e., forward, biotinylated-reverse, and sequencing primers) were designed using the PyroMaker Assay Design 2.0 software (Qiagen, Venlo, Netherlands), and sequences are provided in Supplementary Table S2. Bisulfite-converted LINE-1 DNA fragments were PCR amplified using the PyroMark PCR kit (Qiagen, Venlo, Netherlands). Successful PCR amplification was assessed by TBE electrophoresis at 2% agarose gel as described above, after which the PyroMark Q24 Instrument (Qiagen, Venlo, Netherlands) was used to perform pyrosequencing. Biotinylated PCR products were immobilized on streptavidin-coated Sepharose beads (High Performance, GE Healthcare, Chicago, IL, USA), captured by the PyroMark vacuum Q24 workstation, washed, and denatured. Single-stranded PCR products were then released into a 24-well plate and annealed to the sequencing primer for 2 min at 80 °C. After completion of the pyrosequencing run, results were analyzed using the PyroMark Q24 software (Qiagen, Venlo, The Netherlands).

### 4.12. Infinium Methylation EPIC BeadChip Analysis

Genome-wide DNA methylation was analyzed on the Infinium Methylation EPIC BeadChip platform (Illumina, San Diego, CA, USA) at the Center for Medical Genetics (UZA, University of Antwerp). A total of 500 ng of bisulfite-converted DNA from MM1S and MM1R cells left untreated, treated with 1 µM RSL3, or treated with 2 µM Fer-1 and 1 µM RSL3 was used for whole-genome amplification, enzymatic fragmentation, precipitation, and resuspension as described in the manufacturer’s protocol. EPIC chips were analyzed using the Illumina Hi-Scan system and DNA methylation was measured at 866,836 CpG sites.

DNA methylation data were processed using the minfi (v1.36.0, Massachusetts General Hospital and Harvard Medical School, Boston, MA, USA) [115] and limma (v3.46.0, University of Melbourne, Parkville, Victoria, Australia) [116] R packages as described by Maksimovic et al. [117]. Quality control of all samples occurred by evaluating whether <95% of the CpG probes had a detection *p*-value of < 0.05. Data were subsequently corrected for background signal and normalized using quantile normalization. Finally, cross-reactive and SNP-proximal probes were removed from the data [117]. Normalized data were subsequently analyzed for target selection with a cut-off of 10% differential methylation and FDR < 0.05 (BH-correction). Methylation intensities for each probe are represented as β-values [118]. Heatmap visualization of differentially methylated probes was generated using the heatmap.2 R package (v3.1.1 Tel Aviv University, Tel Aviv, Israel). Pathway enrichment of differentially methylated probes was performed using the Metascape online tool as described above [44]. Methylation data were deposited in the NCBI GEO database (GSE184576).

### 4.13. Global DNA Methylation Analysis by LC-MS/MS

DNA was analyzed by LC-MS/MS as previously described [119]. In short, 0.5 µg of isolated DNA was enzymatically hydrolyzed to individual deoxyribonucleosides by a one-step DNA hydrolysis procedure consisting of a digest mix prepared by adding phosphodiesterase I, alkaline phosphatase, and benzonase nuclease to Tris-HCl buffer (Sigma-Aldrich, Saint Louis, MO, USA). Global genomic DNA (hdyroxy)methylation was measured using ultrapressure liquid chromatography combined with tandem mass spectrometry. Absolute concentrations of cytosine (C), 5-methylcytosine (5-mC), and 5-hydroxymethylcytosine (5-hmC) were calculated by interpolating the results onto a calibration curve. The results are expressed as DNA methylation in percentage (%) (calculated as 5-mC/(5-mC + 5-hmC + C)) and DNA hydroxymethylation (%) (calculated as 5-hmC/(5-mC +5-hmC + C)).

## Figures and Tables

**Table 1:** Overview of RSL3-induced changes in histone PTMs linked to DNA repair and cellular senescence.

Histone PTM or Variant	Expression after RSL3 Treatment	Biological Process	Functional Role	References
H3K79me3	↓	Genome stability	Enriched in heterochromatic centromeric and telomeric regions; prevents spreading of heterochromatin	[48,49,50]
		DNA repair	Controls DNA resection at damaged sites during homologous recombination; crucial in repairing UV-induced DNA damage	[51,52]
H3K18ac	↓	DNA repair	Regulates the expression of antioxidant genes; recruits DNA repair enzymes to damaged sites; regulates expression of nucleotide excision repair-related genes	[53,54,55,56]
		Cellular senescence	H3K18 deacetylation protects cells against mitotic errors and cellular senescence	[57]
		Genome stability	Maintains pericentric hetero-chromatin silencing	[57]
H3K27ac	↓	DNA repair	Regulates the expression of nucleotide excision repair-related genes	[56,58]
		Cellular senescence	Enriched in enhancers of senescence-associated secretory phenotype (SASP) genes	[59,60,61]
H2A.Z	↑	DNA repair	Facilitates chromatin decondensation to allow for loading of DNA repair proteins to DNA breaks	[62,63,64]
		Genome stability	Required for chromosome segregation and cytokinesis; prevents spreading of hetero-chromatin; preserves integrity of centromeres and telomeres	[65,66,67,68]
		Cellular senescence	Overexpression alters regulation of cell cycle and DNA damage repair enzymes and suppresses cellular senescence	[69]
H2A.FY	↑	Cellular senescence	Regulates downstream acetylation to regulate senescence transcription programs	[70,71]
		DNA repair	Alters the kinetics of PAR polymerases during DNA damage responses	[72]

**Table 2:** Common enriched pathways of significant DMP-related genes between RSL3-treated MM1S and MM1R cells.

Common Pathway	GO Term MM1S Cells		GO Term MM1R Cells	
	*GO Term*	*−log p-Value*	*GO Term*	*−log p-Value*
Cell cycle	Negative regulation of cell cycle process	10	DNA replication	5
	Negative regulation of nuclear division	8	Meiotic cell cycle	3.8
	S-phase	4.5	Cell cycle	2.4
	Negative regulation of meiotic nuclear division	3.8	Regulation of transcription involved in G1/S transition of mitotic cell cycle	2.1
Autophagy	Regulation of autophagy	4.4	Autophagy	2.2
Insulin Signaling Pathway	Insulin signaling pathway	3.8	Insulin processing	2.4
			Insulin-like growth factor receptor signaling pathway	2.0
CtBP core complex	CtBP complex	3.7	CtBP core complex	3.9
VEGF Signaling	VEGFA-VEGFR2 signaling pathway	2.3	VEGFA-VEGFR2 Signaling Pathway	4.6
Rett Syndrome	Rett syndrome-causing genes	3.1	Rett syndrome-causing genes	2.5

## Data Availability

The RNA sequencing and BeadChip Array data presented in this study are openly available in the GEO repository (with GSE22309570 and GSE184576, respectively).

## Supplementary Materials

The following are available online at https://www.mdpi.com/article/10.3390/ijms222212234/s1, Figure S1: Relative viability of MM1 cells upon dexamethasone and ferrostatin-1 pre-treatment, Figure S2: Relative viability and lipid peroxidation analysis of MM1R cells treated with RSL3, Figure S3: Gene expression of iron and ferroptosis responsive genes in ferroptotic MM1 cells, Figure S4: Histone extraction optimization from MM1 cells for LC-MS analysis of post-translational modifications, Figure S5: Western blot validation of histone proteomics experiments, Figure S6: Annotated MS/MS spectra of H3 and H2A, Figure S7: Pathway analysis of significant differentially methylated CpG probes in RSL3 MM1S cells, Figure S8: Pathway analysis of significant differentially methylated CpG probes in RSL3 MM1R cells, Table S1: Overview of significantly altered histone PTMs in RSL3-treated MM1 cells, Table S2: Overview of primers used in this study, Table S3: Overview of full-length Western blots.
