# Supplementary material for: Ferroptosis Induction in Multiple Myeloma Cells Triggers DNA Methylation and Histone Modification Changes Associated with Cellular Senescence
Source: Int J Mol Sci. 2021 Nov 12;22(22):12234. doi: 10.3390/ijms222212234 (PMC8618106; doi:10.3390/ijms222212234)
Supplement: Supplementary file 1 [file ijms-22-12234-s001.zip › ijms-1421109-supplementary.pdf]

International Journal of
Molecular Sciences

Supplementary Materials

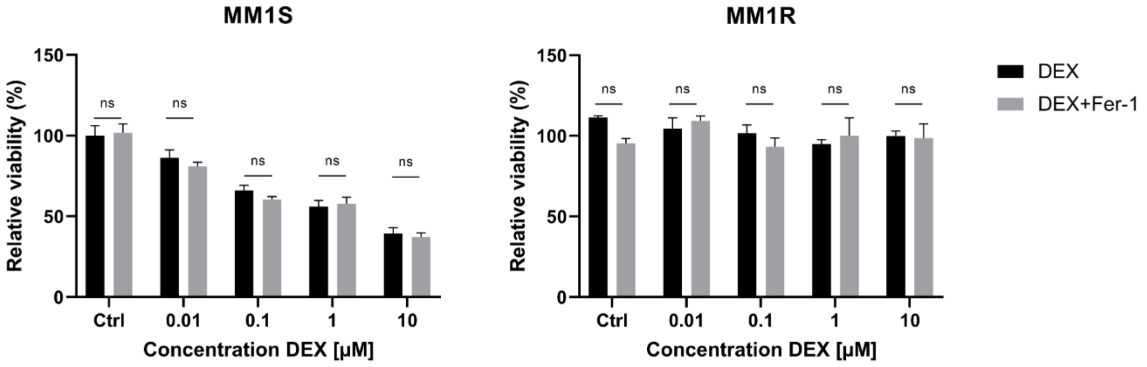

Figure S1. Relative viability of MM1S (left) and MM1R (right) cells upon 24 h exposure to increasing concentrations of dexamethasone (DEX) with or without pre-treatment with ferrostatin-1 (Fer-1). Data are plotted as the mean ± s.d., $n = 3$ biologically independent replicates (ns $p < 0.05$, ANOVA).

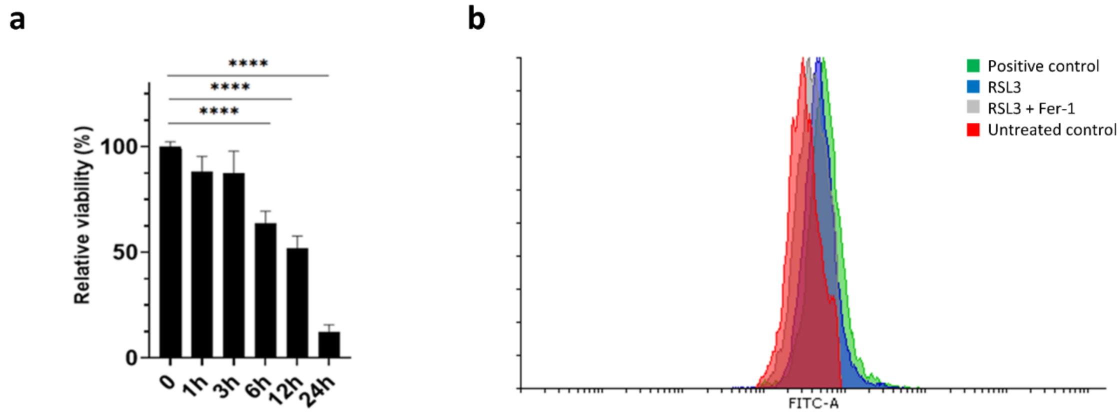

Figure S2. (**a**) Time-dependent relative viability of MM1 cells after 5 µM RSL3 treatment. Data are plotted as the mean ± s.d., $n = 3$ biologically independent replicates (****$p < 0.0001$, ANOVA). (**b**) Flow cytometric analysis of the lipid peroxidation sensor (C11-BODIPY-581/591 dye) on live MM1R cells after 3h treatment with 5µM RSL3 with or without pre-treatment with 2 µM ferrostatin-1 (Fer-1) or 100 µM cumene hydroxide (positive control).

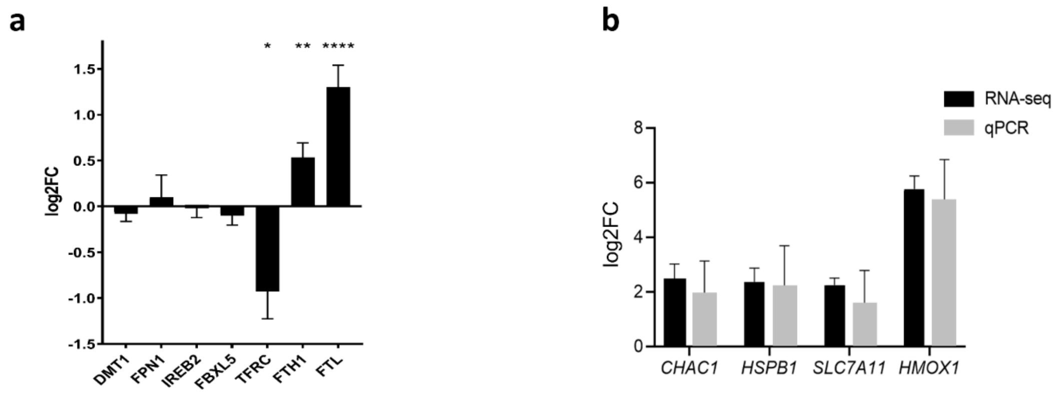

Figure S3. (**a**) LogFC of iron-responsive genes in RSL3-treated MM1 cells versus untreated controls as determined by RNAseq. Data are plotted as the mean logFC ± SEM, n=3 biologically independent replicates per cell line (*$p < 0.05$, **$p < 0.01$, ****$p < 0.0001$, ANOVA). (**b**) Comparison of log2FC of RNAseq and qPCR analysis of 4 ferroptosis-related genes. Data are plotted as the mean ± s.d., $n = 3$ biologically independent replicates per cell line.

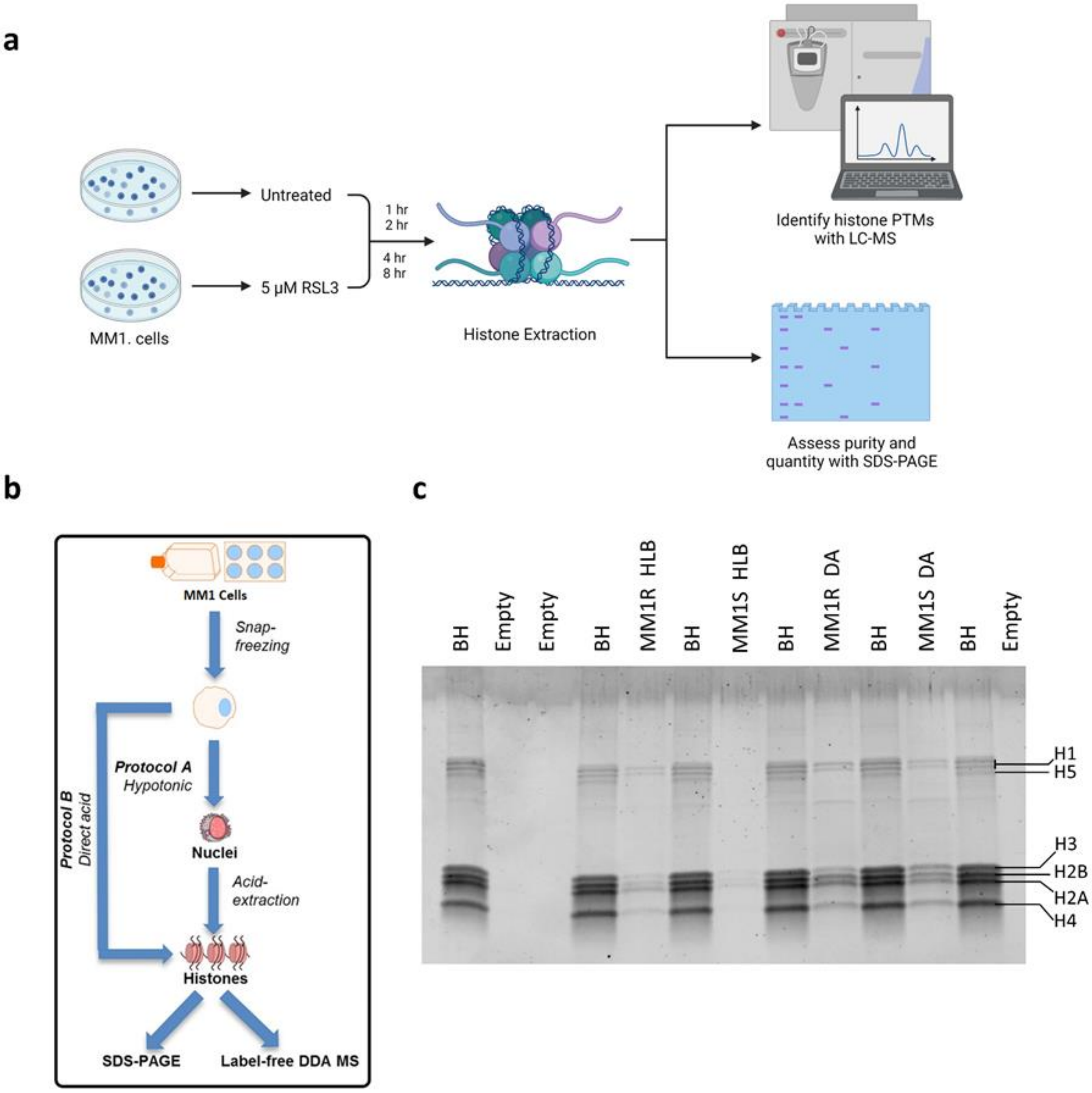

Figure S4. Histone extraction optimization from MM1 cells for LC-MS analysis of post-translational modifications (PTMs). (**a**) Experimental pipeline for identification of histone PTMs with liquid chromatography-mass spectrometry (LC-MS). Prior to LC-MS analysis, purity and quantity of histone extracts is assessed with polyacrylamide gel electrophoresis (PAGE). (**b**) Lysis protocols that were explored for histone extraction from MM1 cells. Adapted from [108]. (**c**) Purity of MM1 histone extracts obtained with either hypotonic lysis buffer (HLB) or direct acid lysis (DA). Extracts from 4 x 10^5 cells were loaded onto a 8–16% gradient gel and compared to a 2 µg bovine histone (BH) commercial standard.

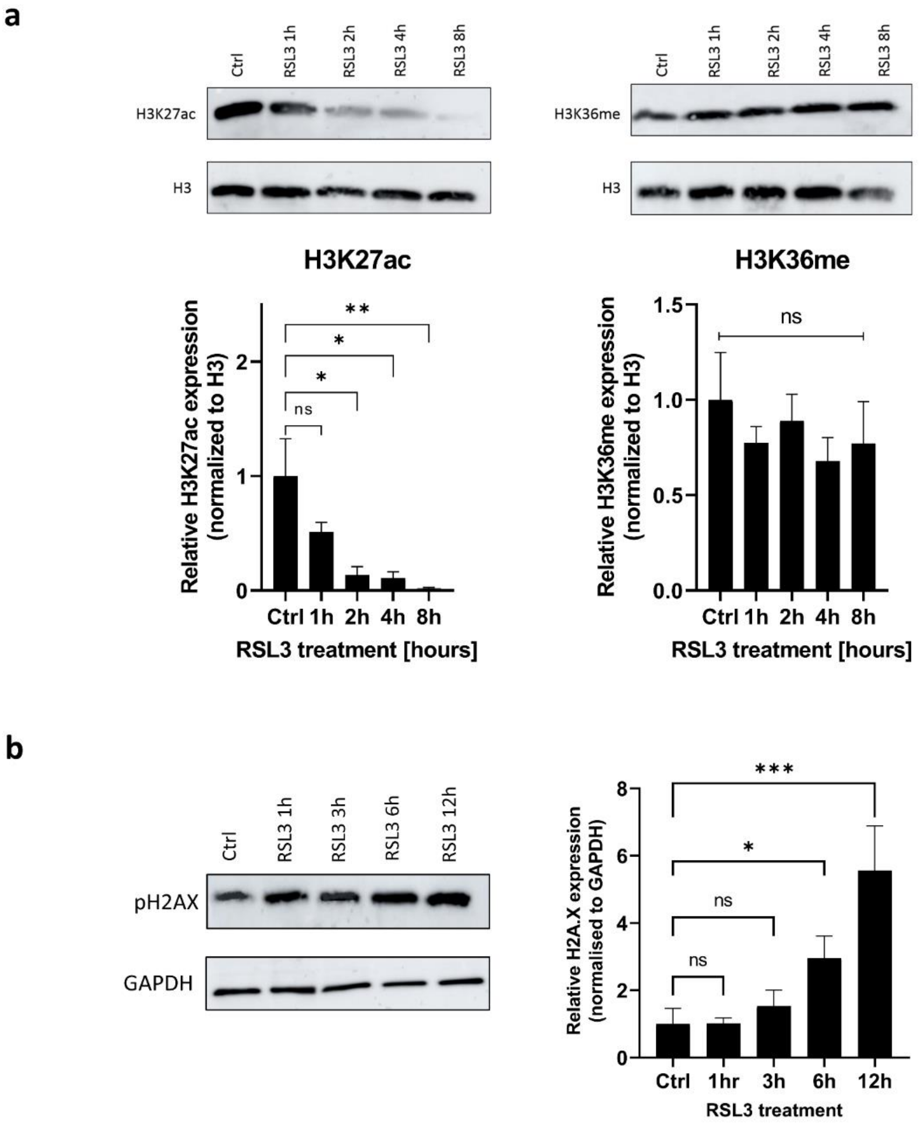

Figure S5. (**a**) Western blot validation of histone proteomics LC-MS data. H3K27ac (left) was significantly upregulated upon RSL3 treatment in MM1 cells while H3K36me3 (right) levels remained unchanged, both in LC-MS and western blot analysis. (**b**) Western blot detection and quantification of pH2AX and GAPDH expression levels after RSL3 treatment in MM1 cells. Data are plotted as the mean ± s.d., $n = 3$ biologically independent replicates. (ns $p > 0.05$, *$p < 0.05$, **$p < 0.01$, ***$p < 0.001$ ANOVA).

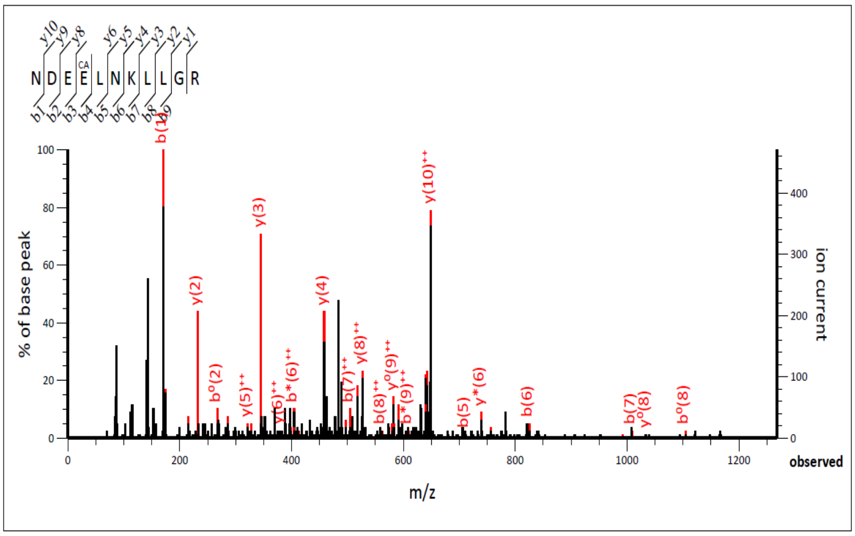

Monoisotopic mass of neutral peptide Mr(calc): 1465.6500
Fixed modifications: Propionyl (K),Propionyl (N-term) (apply to specified residues or termini only)
Variable modifications:
E4 : Cation:Fe[II] (DE)
Ions Score: 24 Expect: 0.0061 (help)

#	b	b^{++}	b^{*}	b^{*++}	b^{0}	b^{0++}	Seq.	y	y^{++}	y^{*}	y^{*++}	y^{0}	y^{0++}	#
1	**171.0764**	86.0418	154.0499	77.5286			N							11
2	**286.1034**	143.5553	**269.0768**	135.0420	**268.0928**	134.5500	D	1296.5881	**648.7977**	1279.5616	**640.2844**	1278.5776	**639.7924**	10
3	**415.1460**	208.0766	**398.1194**	199.5633	397.1354	199.0713	E	1181.5612	**591.2842**	1164.5346	**582.7710**	1163.5506	**582.2789**	9
4	**598.1078**	299.5576	**581.0813**	291.0443	**580.0973**	290.5523	E	1052.5186	**526.7629**	1035.4920	**518.2497**	**1034.5080**	**517.7577**	8
5	**711.1919**	356.0996	694.1654	347.5863	693.1813	347.0943	L	869.5567	435.2820	852.5302	426.7687			7
6	**825.2348**	**413.1211**	808.2083	**404.6078**	**807.2243**	**404.1158**	N	**756.4726**	**378.7400**	**739.4461**	**370.2267**			6
7	**1009.3560**	**505.1816**	**992.3295**	**496.6684**	991.3454	**496.1764**	K	**642.4297**	**321.7185**	625.4032	313.2052			5
8	**1122.4401**	**561.7237**	**1105.4135**	553.2104	**1104.4295**	**552.7184**	L	**458.3085**	**229.6579**	**441.2820**	221.1446			4
9	1235.5241	**618.2657**	1218.4976	**609.7524**	1217.5136	**609.2604**	L	**345.2245**	**173.1159**	**328.1979**	164.6026			3
10	1292.5456	646.7764	1275.5190	638.2632	1274.5350	637.7712	G	**232.1404**	116.5738	**215.1139**	108.0606			2
11							R	**175.1190**	88.0631	**158.0924**	79.5498			1

(a)

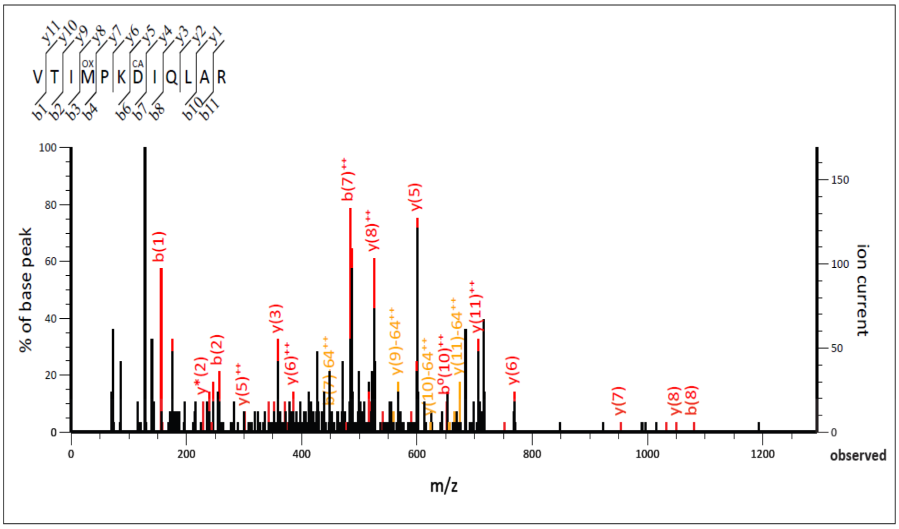

Monoisotopic mass of neutral peptide Mr(calc): 1565.7574
Fixed modifications: Propionyl (K),Propionyl (N-term) (apply to specified residues or termini only)
Variable modifications:
M4 : Oxidation (M), with neutral losses 0.0000(shown in table), 63.9983
D7 : Cation:Fe[II] (DE)
Ions Score: 39 Expect: 0.00047 (help)

#	b	b^{++}	b*	b^{*++}	b^{0}	b^{0++}	Seq.	y	y^{++}	y*	y^{*++}	y^{0}	y^{0++}	#
1	156.1019	78.5546					V							12
2	257.1496	129.0784			239.1390	120.0731	T	1411.6701	706.3387	1394.6435	697.8254	1393.6595	697.3334	11
3	370.2336	185.6205			352.2231	176.6152	I	1310.6224	655.8148	1293.5959	647.3016	1292.6118	646.8096	10
4	517.2690	259.1382			499.2585	250.1329	M	1197.5383	599.2728	1180.5118	590.7595	1179.5278	590.2675	9
5	614.3218	307.6645			596.3112	298.6593	P	1050.5029	525.7551	1033.4764	517.2418	1032.4924	516.7498	8
6	798.4430	399.7251	781.4164	391.2119	780.4324	390.7199	K	953.4502	477.2287	936.4236	468.7155	935.4396	468.2234	7
7	967.3892	484.1982	950.3627	475.6850	949.3787	475.1930	D	769.3290	385.1681	752.3025	376.6549	751.3184	376.1629	6
8	1080.4733	540.7403	1063.4467	532.2270	1062.4627	531.7350	I	600.3828	300.6950	583.3562	292.1817			5
9	1208.5319	604.7696	1191.5053	596.2563	1190.5213	595.7643	Q	487.2987	244.1530	470.2722	235.6397			4
10	1321.6159	661.3116	1304.5894	652.7983	1303.6054	652.3063	L	359.2401	180.1237	342.2136	171.6104			3
11	1392.6530	696.8302	1375.6265	688.3169	1374.6425	687.8249	A	246.1561	123.5817	229.1295	115.0684			2
12							R	175.1190	88.0631	158.0924	79.5498			1

(**b**)

Figure S6. Annotated MS/MS spectra of H3 (**a**) and H2A (**b**) proteins isolated from RSL3-treated MM1 cells. Annotated spectra indicate Fe^{2+} binding to glutamate (E) and aspartate (D) residues of H2A and H3.

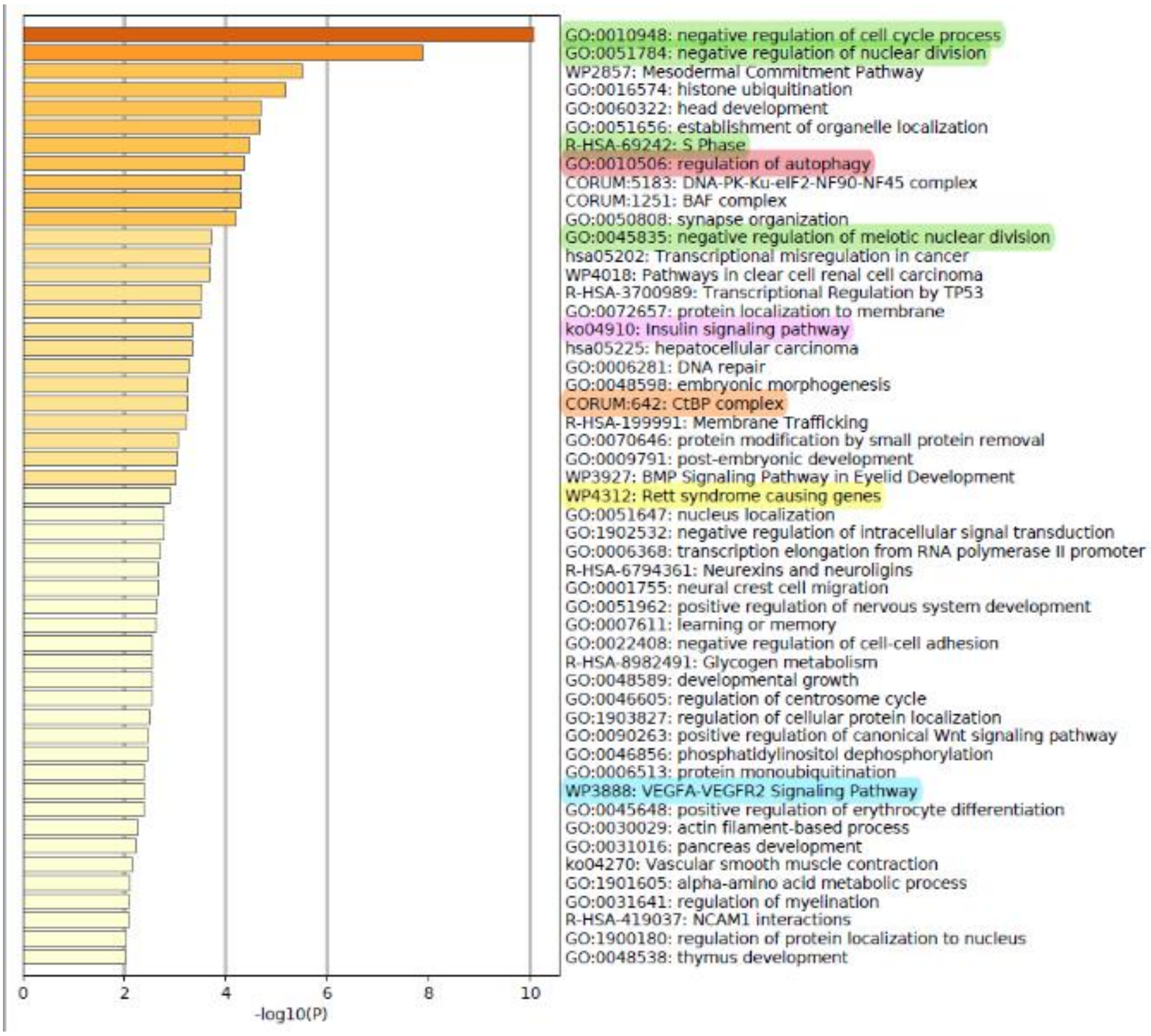

Figure S7. Metascape pathway enrichment of significantly differentially methylated CpG probes (FDR <0.05 & $\Delta\beta$ differences > 0.1) in RSL3-treated **MM1S** cells compared to Ferrostatin-1 pre-treated, RSL3-treated MM1S cells. Terms marked in color are common pathways between MM1S and MM1R cells (see Figure S6).

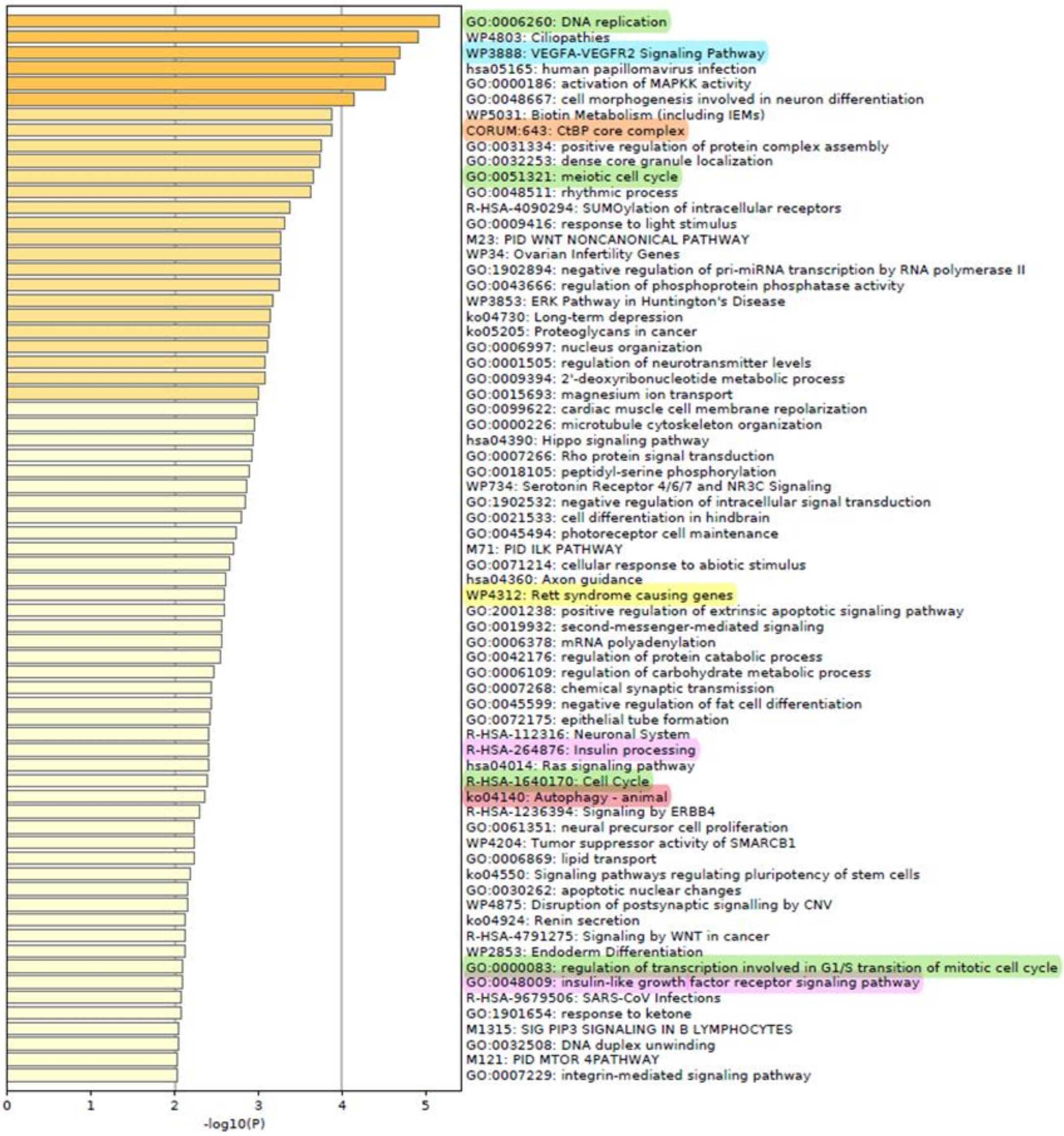

Figure S8: Metascape pathway enrichment of significantly differentially methylated CpG probes (FDR <0.05 & Δβ differences > 0.1) in RSL3-treated MM1R cells compared to Ferrostatin-1 pre-treated, RSL3-treated MM1S cells. Terms marked in color are common pathways between MM1S and MM1R cells (see Figure S5).

Table S1. Overview of significantly altered histone PTMs in RSL3-treated MM1 cells compared to untreated controls.

Histone	PTM	Sequence	Log2FC				*p*-Value			
			1 h	2 h	4 h	8 h	1 h	2 h	4 h	8 h
**H2A**	E57Fe	VGA-GAPVYMAAVLEYLTAEILELAGNAAR	0,20	0,61	0,69	0,56	$3,70 \times 10^{-1}$	$1,46 \times 10^{-3}$	$1,75 \times 10^{-4}$	$5,90 \times 10^{-3}$
	R89Cit	HLQLAIRNDEELNKLLGR	−0,15	−0,63	−0,47	−0,53	$4,51 \times 10^{-1}$	$1,27 \times 10^{-2}$	$5,28 \times 10^{-2}$	$3,54 \times 10^{-2}$
	E93Fe	NDEELNKLLGR	0,32	0,63	0,80	0,90	$1,21 \times 10^{-1}$	$6,33 \times 10^{-4}$	$1,93 \times 10^{-5}$	$1,84 \times 10^{-4}$
	K96Ac	HLQLAIRNDEELNKLLGK	0,52	2,26	2,86	1,93	$2,99 \times 10^{-1}$	$5,55 \times 10^{-3}$	$1,37 \times 10^{-3}$	$1,17 \times 10^{-2}$
	K100Ac	HLQLAIRNDEELNKLLGK	0,52	2,26	2,86	1,93	$2,99 \times 10^{-1}$	$5,55 \times 10^{-3}$	$1,37 \times 10^{-3}$	$1,17 \times 10^{-2}$
	K100But	HLQLAIRNDEELNKLLGK	0,52	2,26	2,86	1,93	$2,99 \times 10^{-1}$	$5,55 \times 10^{-3}$	$1,37 \times 10^{-3}$	$1,17 \times 10^{-2}$
**H2A.Z**	K8Ac	AGGKAGKDSGKAKA-KAVSR	0,08	−0,14	−0,23	−0,61	$6,84 \times 10^{-1}$	$4,78 \times 10^{-1}$	$2,78 \times 10^{-1}$	$1,68 \times 10^{-2}$
	K14Ac	AGGKAGKDSGKAKA-KAVSR	−0,77	−2,49	−2,32	−2,08	$2,29 \times 10^{-1}$	$1,36 \times 10^{-2}$	$1,61 \times 10^{-2}$	$2,81 \times 10^{-2}$
	K16Suc	AGGKAGKDSGKAKA-KAVSR	−0,77	−2,49	−2,32	−2,08	$2,29 \times 10^{-1}$	$1,36 \times 10^{-2}$	$1,61 \times 10^{-2}$	$2,81 \times 10^{-2}$
	K16Hib	AGGKAGKDSGKAKA-KAVSR	−0,43	−3,28	−2,15	−2,15	$5,66 \times 10^{-1}$	$3,18 \times 10^{-2}$	$6,74 \times 10^{-2}$	$8,32 \times 10^{-2}$
	K102Hib	HLQLAIRGDEELDSLIKATI-AGGGVIPHIHKSLIGKKGQQKTA	−0,08	−0,56	−0,98	−0,84	$8,10 \times 10^{-1}$	$1,21 \times 10^{-1}$	$1,94 \times 10^{-2}$	$4,04 \times 10^{-2}$
	K120Cro	HLQLAIRGDEELDSLIKATI-AGGGVIPHIHKSLIGKKGQQKTA	−0,08	−0,56	−0,98	−0,84	$8,10 \times 10^{-1}$	$1,21 \times 10^{-1}$	$1,94 \times 10^{-2}$	$4,04 \times 10^{-2}$
	K121Me3	HLQLAIRGDEELDSLIKATI-AGGGVIPHIHKSLIGKKGQQKTA	−0,08	−0,56	−0,98	−0,84	$8,10 \times 10^{-1}$	$1,21 \times 10^{-1}$	$1,94 \times 10^{-2}$	$4,04 \times 10^{-2}$
	K121But	HLQLAIRGDEELDSLIKATI-AGGGVIPHIHKSLIGKKGQQKTA	−0,20	−1,55	−0,87	−0,63	$5,91 \times 10^{-1}$	$2,93 \times 10^{-3}$	$4,95 \times 10^{-2}$	$0,14 \times 10^{-1}$
	K125Me3	HLQLAIRGDEELDSLIKATI-AGGGVIPHIHKSLIGKKGQQKTA	−0,08	−0,56	−0,98	−0,84	$8,10 \times 10^{-1}$	$1,21 \times 10^{-1}$	$1,94 \times 10^{-2}$	$4,04 \times 10^{-2}$
**H2A.FY**	K33But	YIKKGHPKYR	−0,04	−0,90	−1,32	0,56	$8,36 \times 10^{-1}$	$1,72 \times 10^{-3}$	$5,90 \times 10^{-5}$	$0,38 \times 10^{-1}$
	K34Me3	YIKKGHPKYR	−0,04	−0,90	−1,32	0,56	$8,36 \times 10^{-1}$	$1,72 \times 10^{-3}$	$5,90 \times 10^{-5}$	$0,38 \times 10^{-1}$
	R79Me	VTPRHILLA-VANDEELNQLLKGVTIASGGVLPNIHPELLAKKR	0,09	0,55	0,62	0,73	$8,19 \times 10^{-1}$	$9,05 \times 10^{-2}$	$7,74 \times 10^{-2}$	$2,34 \times 10^{-2}$
**H3**	K18Ac	KQLATKAAR	−0,61	−5,01	−2,99	−3,30	$4,03 \times 10^{-1}$	$1,67 \times 10^{-2}$	$3,11 \times 10^{-2}$	$3,31 \times 10^{-2}$

	K27Ac	KSAPATGGVKKPHR	−0,40	−0,56	−0,72	−0,56	$1,38 \times 10^{-1}$	$6,21 \times 10^{-2}$	$2,19 \times 10^{-2}$	$8,62 \times 10^{-2}$
	K37Me3	KSAPATGGVKKPHR	−0,91	−2,79	−2,80	−2,03	$7,09 \times 10^{-2}$	$8,88 \times 10^{-4}$	$9,00 \times 10^{-4}$	$5,21 \times 10^{-3}$
	K37Bu	KSAPSTGGVKKPHR	−0,11	0,19	0,17	0,34	$3,94 \times 10^{-1}$	$1,27 \times 10^{-1}$	$1,91 \times 10^{-1}$	$2,02 \times 10^{-3}$
	R40Cit	KSAPATGGVKKPHR	0,12	0,45	0,31	0,66	$4,46 \times 10^{-1}$	$5,00 \times 10^{-4}$	$6,95 \times 10^{-2}$	$3,99 \times 10^{-5}$
	D77Fe	EIAQDFKTDLR	0,25	0,73	0,78	0,62	$2,73 \times 10^{-1}$	$1,79 \times 10^{-4}$	$6,56 \times 10^{-5}$	$5,31 \times 10^{-3}$
	K79But	EIAQDFKTDLR	0,04	0,25	0,11	0,74	$7,54 \times 10^{-1}$	$4,46 \times 10^{-2}$	$3,66 \times 10^{-1}$	$2,48 \times 10^{-4}$
	K79Me3	EIAQDFKTDLR	−0,27	−0,84	−0,80	-1,07	$4,76 \times 10^{-2}$	$1,14 \times 10^{-6}$	$2,40 \times 10^{-6}$	$1,45 \times 10^{-7}$
	R83Cit	EIAQDFKTDLR	0,01	0,22	−0,08	0,94	$9,72 \times 10^{-1}$	$3,56 \times 10^{-1}$	$7,61 \times 10^{-1}$	$3,99 \times 10^{-3}$
	M97Ox	VTIMPKDIQLAR	0,57	1,43	1,39	1,32	$1,36 \times 10^{-2}$	$2,49 \times 10^{-2}$	$2,46 \times 10^{-2}$	$2,48 \times 10^{-2}$
	D100Fe	VTIMPKDIQLAR	0,56	1,57	2,25	1,38	$1,43 \times 10^{-1}$	$2,83 \times 10^{-1}$	$0,35 \times 10^{-1}$	$0,27 \times 10^{-1}$
**H4**	R17Me	GKGGKGLGKGGAKRHR	−0,17	0,47	0,02	0,53	$5,09 \times 10^{-1}$	$2,53 \times 10^{-2}$	$9,31 \times 10^{-1}$	$1,02 \times 10^{-2}$
	E63Fe	GVLKVFLENVIR	0,37	0,88	0,96	0,60	$4,50 \times 10^{-1}$	$2,32 \times 10^{-2}$	$1,51 \times 10^{-2}$	$0,18 \times 10^{-1}$
	M84Ox	KTVTAMDVVYALKR	0,12	0,29	0,29	0,28	$2,46 \times 10^{-1}$	$3,92 \times 10^{-3}$	$3,81 \times 10^{-3}$	$7,12 \times 10^{-3}$
	D85Fe	KTVTAMDVVYALKR	0,09	0,57	0,54	0,52	$5,36 \times 10^{-1}$	$1,56 \times 10^{-5}$	$1,23 \times 10^{-4}$	$4,66 \times 10^{-4}$

Table S2. Overview of primers used in this study.

Target		Primer Sequence	Gene Accession Number
		qPCR primers	
CHAC1	Forward<br>Reverse	ATG CCT GGC CGT GTG G<br>GCT TAC CTG CTC CCC TTG C	ENSG00000128965
HSPB1	Forward<br>Reverse	AGG ATG GCG TGG TGG AGA T<br>GAT GTA GCC ATG CTC GTC CTG	ENSG00000106211
SLC7A11	Forward<br>Reverse	CAC ATG CCT CTT CAT GGT TG<br>AGT GAT GAC GAA GCC AAT CC	ENSG00000151012
HMOX1	Forward<br>Reverse	CCA GCG GGC CAG CAA CAA AGT GC<br>AAG CCT TCA GTG CCC ACG GTA AGG	ENSG00000100292
ACTB	Forward<br>Reverse	CTG GAA CGG TGA AGG TGA CA<br>AAG GGA CTTC CTG TAA CAA TGC A	ENSG00000075624
NR4A2	Forward<br>Reverse	GGT CCC TTT TGC CTG TCC A<br>TGG CTT CAG CCG AGT TAC AG	ENSG00000153234
		Methylation-specific PCR primers	
SALL3	Forward	GTT TGG GTT TGG TTT TTG TT	ENSG00000263310

	Reverse	ACC CTT TAC CAA TCT CTT AAC TTT C
	Pyrosequencing primers	
LINE-1	Forward	TTT TTT GAG TTA GGT GTG GG
	Reverse	TCT CAC TAA AAA ATA CCA AAC AA
	Sequencing	GGG TGG GAG TGA T

Table S3. Original images western blot analysis.

<table>
<tr><th>Figure</th><th>Blot(s) with molecular marker</th></tr>
<tr><td>7: Western blot detection and quantification of NR4A2 and GAPDH protein expression levels after RSL3 treatment in MM1 cells</td><td>α-NR4A2 (70 kDa) and α-GAPDH (37 kDa)
marker: Benchmark Pre-stained protein standard (Thermo, #10748-010)

α-NR4A2
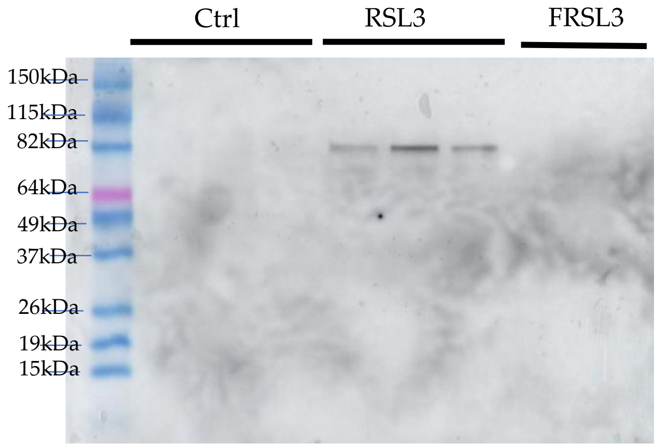

α-GAPDH
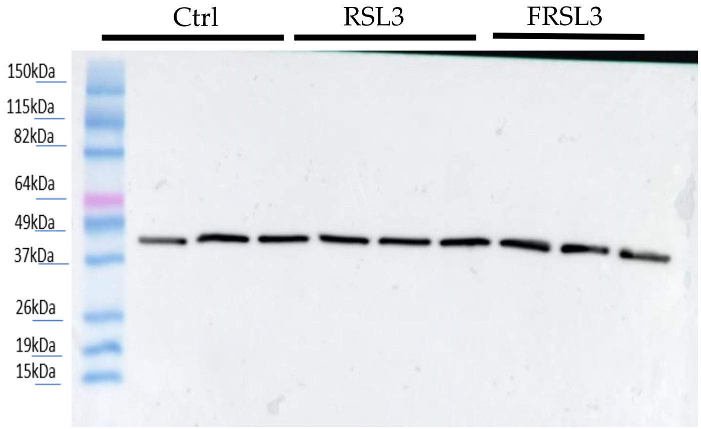
</td></tr>
<tr><td>S5a: Western blot detection and quantification of H3K27ac, H3K36me, and H3 protein expression levels after RSL3 treatment in MM1R cells</td><td>α-H3K27ac (15 kDa), and α-H3 (11 kDa)
marker: Precision Plus Protein All Blue prestained protein standard (Bio-Rad, #1610373)

α-H3K27ac   Ctrl   RSL3 1h   RSL3 2h   RSL3 4h   RSL3 8h</td></tr>
</table>

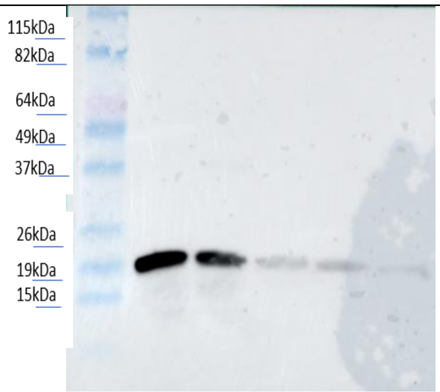

α-H3

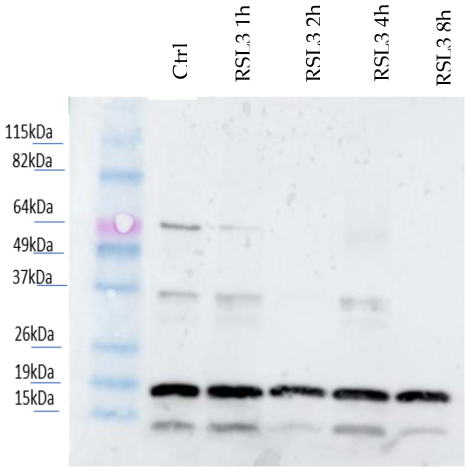

α-H3K36me (full length kDa, and α-H3 (11 kDa)
marker: Precision Plus Protein All Blue prestained protein standard (Bio-Rad, #1610373)

α-H3K36me

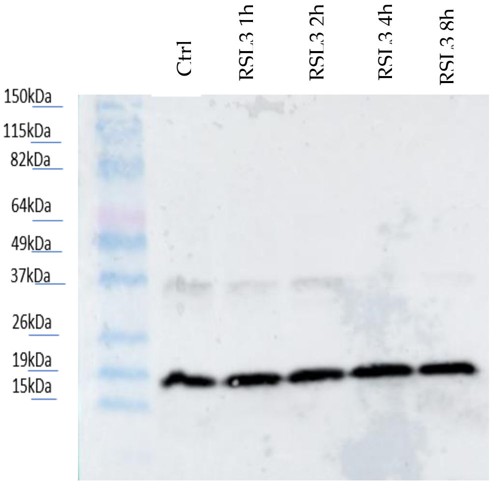

α-H3

Ctrl RSL3 1h RSL3 2h RSL3 4h RSL3 8h

	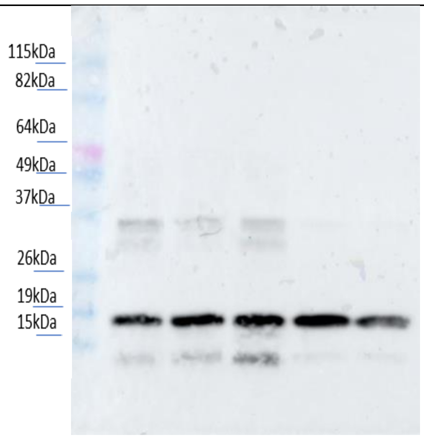
**S5b:** Western blot detection and quantification of pH2AX and H3 protein expression levels after RSL3 treatment in MM1R cells	α-pH2AX (15 kDa), and α-GAPDH (37 kDa)<br>*marker:* Precision Plus Protein All Blue prestained protein standard (Bio-Rad, #1610373)<br><br>α-pH2AX<br>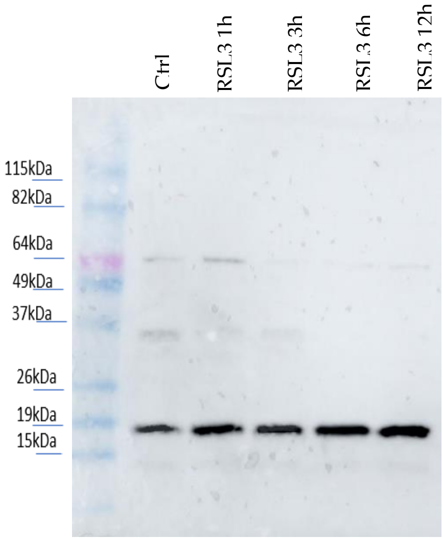 <br>α-GAPDH<br>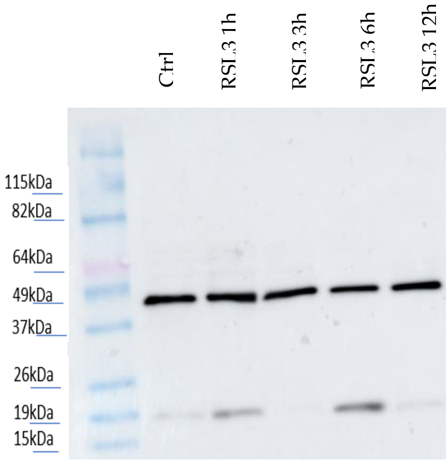
